# Characterization, Recombinant Production and Structure-Function Analysis of NvCI, A Picomolar Metallocarboxypeptidase Inhibitor from the Marine Snail *Nerita versicolor*

**DOI:** 10.3390/md17090511

**Published:** 2019-08-29

**Authors:** Giovanni Covaleda-Cortés, Martha Hernández, Sebastián Alejandro Trejo, Manuel Mansur, Sergi Rodríguez-Calado, Javier García-Pardo, Julia Lorenzo, Josep Vendrell, María Ángeles Chávez, Maday Alonso-del-Rivero, Francesc Xavier Avilés

**Affiliations:** 1 Institute of Biotechnology and Biomedicine and Departament of Biochemistry and Molecular Biology, Universitat Autònoma de Barcelona, 08193 Bellaterra (Barcelona), Spain; 2 Faculty of Forestry Science, Biotechnology Center, Universidad de Concepción, Victoria 631, Barrio Universitario, 2407 Concepción, Chile; 3 Center for Protein Research, Faculty of Biology, Universidad de la Habana, 10400 La Habana, Cuba

**Keywords:** *Nerita versicolor*, proteinaceous inhibitor, recombinant production, carboxypeptidase, picomolar inhibition, biotechnological and biomedical applications

## Abstract

A very powerful proteinaceous inhibitor of metallocarboxypeptidases has been isolated from the marine snail *Nerita versicolor* and characterized in depth. The most abundant of four, very similar isoforms, NvCla, was taken as reference and N-terminally sequenced to obtain a 372-nucleotide band coding for the protein cDNA. The mature protein contains 53 residues and three disulphide bonds. NvCIa and the other isoforms show an exceptionally high inhibitory capacity of around 1.8 pM for human Carboxypeptidase A1 (hCPA1) and for other A-like members of the M14 CPA subfamily, whereas a twofold decrease in inhibitory potency is observed for carboxypeptidase B-like members as hCPB and hTAFIa. A recombinant form, rNvCI, was produced in high yield and HPLC, mass spectrometry and spectroscopic analyses by CD and NMR indicated its homogeneous, compact and thermally resistant nature. Using antibodies raised with rNvCI and histochemical analyses, a preferential distribution of the inhibitor in the surface regions of the animal body was observed, particularly nearby the open entrance of the shell and gut, suggesting its involvement in biological defense mechanisms. The properties of this strong, small and stable inhibitor of metallocarboxypeptidases envisage potentialities for its direct applicability, as well as leading or minimized forms, in biotechnological/biomedical uses.

## 1. Introduction

Unravelling the detailed molecular determinants by which an enzyme, its substrates or inhibitors follow a precise mechanism of recognition and action is a key step that may drive a better understanding of the behavior of similar molecules and to the design of novel synthetic structures that facilitate biotechnological applicability. Both aims have been addressed and achieved for many proteolytic enzymes and ligands because of their wide distribution in living organisms and their involvement in key functions and distresses, including metallocarboxypeptidases (MCPs or CPs) [1,2]. However, although some members of this type of proteases are among the first enzymes for which the basic structure-function features were solved [3], the great number of genomic and proteomic variants of them, with more than 30 members described nowadays [4,5], demand further characterization and the search for new molecular modulators. 

The proteinaceous inhibitors of CPs are particularly valuable molecules because of their binding in a substrate-like manner to the enzyme. In several instances this results in an initial recognition and C-terminal cleavage of the inhibitor, followed by the establishment of a very stable complex with the enzyme, which facilitates structural and mechanistic analyses [5,6,7]. This has been the case for few of such proteinaceous inhibitors: those from potato (PCI) [8], tomato [9], the intestinal parasite *Ascaris suum* (ACI) [10], the medical leech *Hirudo medicinalis* (LCI) [11], the ticks *Riphicephalus bursa* (TCI) [12] and *Haemaphysalis longicornis* (HITCI) [13] and the mollusk *Nerita versicolor* [14]. Also, the variants from rat and human tissues (Latexin) [15]. However, the latter case, as well that recently characterized from the marine annelid *Sabellastarte magnifica* (SmCI) [16], differ from the former in being much larger (19–22 kDa vs. 5–8 kDa) and displaying a quite different inhibitory mechanism.

Interestingly, most of the above mentioned examples, displayed a remarkable inhibitory capability, with *K*_i_ values in the nanomolar range (about 1–40 nM), but failed to reach the picomolar level that has been described for certain proteinaceous inhibitors of other proteolytic enzymes, like serine- or cysteine-proteases [17,18]. The small size of the C-terminal inhibitory tails of the inhibitors mentioned above as compared to the extended inhibitory regions of serine- or cysteine-protease inhibitors, the number of stabilizing interactions or the entropic constraints needed to anchor a free-moving tail into the enzyme active site may be some of the reasons that could account for the apparent lower performance of CP inhibitors.

Some of these questions might be answered with the detailed analysis of the potent proteinaceous CP inhibitor characterized in depth in this report, isolated from the body of the marine snail *Nerita versicolor*. It is a small form (with a mass around 5945 Da and 53 residues) stabilized by three disulphides, with the shortest C-tail reported for such a kind of inhibitors (only two residues), but displaying the highest inhibitory power described until now, with *K*_i_ in the 1-10 pM and 0.1–0.9 nM ranges for several mammalian carboxypeptidases A and B, respectively. This unusually tight inhibitor is found in several isoforms in the snail and here we report the isolation and identification of four of them, the biochemical and cellular characterization of a main isoform and the development of an efficient recombinant production method in yeast. Also, the location of the inhibitor in the body of the snail and its internalization when added to a given cell culture, among other features. The knowledge of its properties might provide both a further understanding of the limits in the inhibition mechanism and specificities of CPs and, on applied grounds, a basis for a better redesign or the generation of synthetic inhibitors for practical uses.

## 2. Results

### 2.1. Isolation and Purification of NvCI

**Figure 1:**
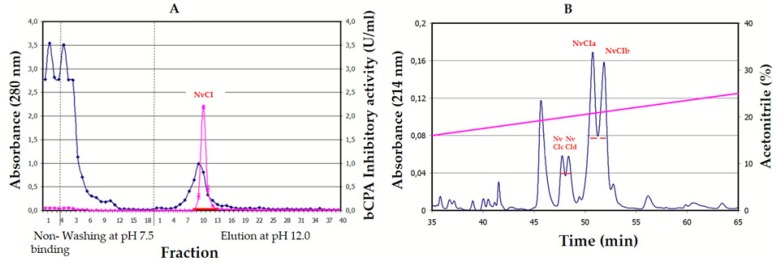
Purification process of natural NvCI. (**A**) Affinity chromatography of the heat-treated extract of *N. versicolor* on a CPA-glyoxal Sepharose^®^ CL-4B column. bCPA1 inhibitory activity is indicated as a grey trace marked with triangles. (**B**) RP-HPLC of the purified NvCI fraction identified with a horizontal line in the affinity chromatography profile. Subfractions NvCIa, NvCIb, NvCIc and NvCId are identified over the profile.

The purification process of natural NvCI from heating-pre-treated snail aqueous extracts involved two chromatographic steps: an affinity chromatography on a CPA-glyoxal Sepharose^®^ 4B CL column and a reverse phase chromatography on a C4 column (Figure 1). The first step allowed to recover 150.6% and 152.5% of the initial inhibitory activity found at the initial aqueous extract and at the heat-treated extract, respectively, suggesting that the inhibitor is not fully free in such initial media. The second step gave rise to several peaks with inhibitory activity, particularly two major (NvCIa, NvCIb) and two minor ones (NvCIc and NvCId) that were subsequently confirmed to be isoforms of the main component, NvCIa. NvCIa, taken as reference for this work, showed a modest 25.7% recovery from reversed-phase high-pressure liquid chromatography (RP-HPLC) regarding the bCPA1 inhibitory activity although its specific activity increased 4444.2 and 87.6-fold relative to the initial extract and the fraction eluted from the affinity chromatography, respectively.

Matrix-Assisted Laser Desorption/Ionization-Time-of-Flight (MALDI-TOF) Mass Spectrometry (MS) analysis of the four RP-HPLC peaks confirmed the purity of each sample. Single peaks at molecular masses of 5946.0, 5959.8, 5798.3 and 5812.5 Da, corresponding to NvCIa, NvCIb, NvCIc and NvCId, respectively, were visualized in the MS spectra (Figure S1). Automated N-terminal Edman degradation of the four species resulted in identical sequences for all of them with the exception made that NvCIc and NvCId lack the N-terminal Phe. In addition, all these isoforms displayed practically the same inhibitory capability against bCPA1. Although the primary structure of all four isoforms of NvCI has been analyzed in the present work (see later on), only the major NvCIa form was selected for further recombinant production and structure-function detailed characterization. Generically, this form is referred to as NvCI.

### 2.2. Primary Sequence of NvCI

**Figure 2:**
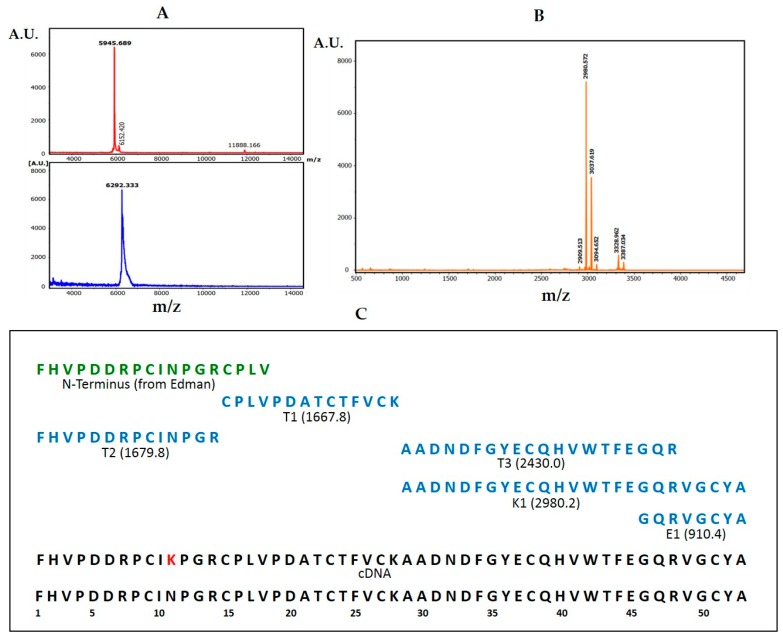
Determination of the amino acid sequence of NvCI by MALDI-TOF MS. (**A**) Top: MALDI-TOF MS spectrum of NvCI; bottom: MALDI-TOF MS spectrum after reduction and S-carbamidomethylation of NvCI. (**B**) MALDI-TOF spectrum of NvCI after enzymatic digestion with Lys-C. (**C**) Complete amino acid sequence of NvCI. The N-terminus sequence (green) was obtained by Edman degradation of the entire molecule. The enzyme used for each digestion (blue) is indicated with letters: T, trypsin; K endoproteinase-Lys-C; and E endoproteinase Glu-C. The theoretical molecular mass of each peptide is shown in parentheses. The two bottom boxes contain the complete amino acid sequence of NvCIa and NvCIb, respectively. The lysine residue at the 11th position (in red) represents the difference found in the NvCIb isoform.

Automated Edman degradation allowed the identification of the first 18 residues of NvCI, with two cysteines included in such region. Comparison of the native and the reduced and S-carbamidomethylated protein by MALDI-TOF MS analysis, indicated that it contains a total of six cysteines involved in disulphide bonds (Figure 2A). Fragmentation of the protein with Lys-C (Figure 2B), Glu-C and trypsin, followed by peptide isolation by RP-HPLC, and analysis by both Edman degradation of peptides and CID-MALDI.MS of the longest C-terminal fragment, allowed deriving the whole amino acid sequence of NvCI. As an example, Figure S2 shows the MALDI MS/MS analysis of one of the peptides obtained from Lys-C fragmentation.

**Figure 3:**
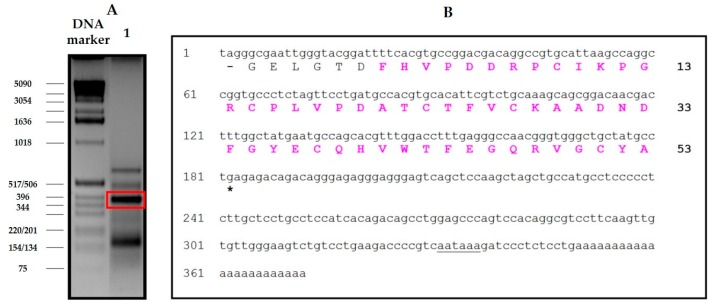
Cloning and sequencing of cDNA encoding NvCI. (**A**) Analysis of PCR products in 2% *w/v* agarose gel. Lane 1: PCR products obtained using the template cDNA from the body of *N. versicolor*. The band marked with a box represents the cDNA encoding NvCI. (**B**) cDNA consensus sequence of the gene encoding NvCI. The “tga” stop codon is marked with an asterisk in the Figure (*), and the canonical polyadenylation signal sequence “aataaa” is underlined.

To facilitate the proper alignment of the derived peptide sequences, and to get further molecular information, the cDNA of NvCI was cloned and sequenced. In short, using semi-degenerated primers from the N-terminal sequence of NvCI and 3′-RACE-PCR, a 372 nucleotides band of cDNA was generated, purified and sequenced (Figure 3). The captured sequence corresponded to the a+b isoforms. It encoded a protein sequence of 53 residues that fully aligned with the one derived directly from the natural protein. The cDNA ended with a 23-residues long polyA stretch. A double read in the alignment was at the 11th protein residue position, where a N→K substitution was detected, which fits exactly with the difference in mass between the NvCIa and NvCIb isoforms, giving support to the occurrence of both variants in the *Nerita versicolor* snail. 

The derived complete primary sequence of NvCI (NvCIa isoform) contains 53 amino acid residues, with Phe and Ala at the N- and C-ends, respectively, and with 6 Cys residues distributed from positions 9 to 51 (Figure 2C). Given that all cysteines are unreactive to alkylation reagents and supposedly involved in intrachain disulphide bonds, it can be assumed that the protein presents two tails, of eight and two residues at the N- and C-terminus, respectively. Analysis of the amino acid sequence of NvCI by the Sequence Editor software (Bruker Daltonics, GE), indicates a theoretical mass of 5944.6 Da for the Asn-containing form NvCIa, revealing also that the MALDI-TOF MS signal displayed for the natural form (5946 Da) corresponds to the protonated molecular ion (M + H)^+^. The amino acid sequence of NvCI has been annotated in the UniProt database with accession number P86912.

MALDI-TOF MS tryptic peptide fingerprints from the RP-HPLC purified forms were obtained to further clarify the amino acid sequences of the different isoforms and back the previous sequencing results (Figure S3), confirming that the less represented forms, NvCIc and NvCId, lack the N-terminal Phe residues as compared to NvCIa and NvCIb forms, respectively, and that both pairs contain the previously detected Asn/Lys exchange. Overall, results point up to a very high sequence identity among the four NvCI isoforms, with only one residue difference, either by substitution or by absence of the N-terminal Phe in the a/b or in a/c and b/d isoforms, respectively.

### 2.3. Production and Characterization of the Recombinant Form of NvCI

**Figure 4:**
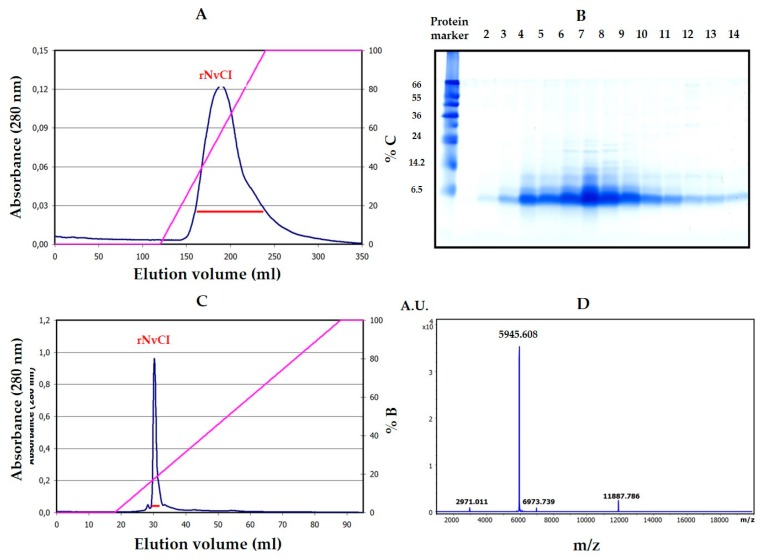
Purification process of recombinant NvCI. (**A**) Weak cation exchange chromatography of the rNvCI fermentation supernatant. (**B**) Tris-tricine SDS-PAGE of the cation exchange chromatography profile. Lane 1: elution fraction obtained at elution volume V_e_ = 150 ml. Subsequent fractions (lanes 2 to 14) were collected at every additional 5 ml V_e_. (**C**) Weak anion exchange chromatography of the elution peak from CM CEC* on a TSK-GEL^®^ DEAE-5PW column. (**D**) MALDI-TOF MS of the rNvCI peak obtained from the TSK^®^-DEAE weak anion exchange column.

In order to obtain a large supply of NvCI (also called NvCIa) and facilitate further characterization and applicative purposes, a recombinant/bioreactor-based DNA approach on the methylotrophic *Pichia pastoris* system was developed with Zeocin hyper-resistant *P. pastoris* transformants. 330 mg/L of inhibitor were obtained as a final purification yield (see Methods). Figure 4 summarizes the steps of the purification process. MS analyses of the pure, carbamidomethylated and trypsin-treated recombinant protein were also carried out to compare it with the natural form (Figure S4). The specific inhibitory activity of the purified protein was 274.8 U/mg. The procedure involved a two-step chromatographic purification with a particular yield of 27%, a point which would require further improvement, but still enough to produce hundreds of mgs of pure and active protein. Double titration of rNvCI inhibitory capability with the alternative inhibitor Tick CP inhibitor (TCI) on bCPA and hCPA1 showed a 95/98% inhibitory capability, and the same picomolar level *K*_i_ than the native protein, an indication of its identity, native state and purity. This was confirmed by trypsin fingerprinting on MALDI-TOF MS (results not shown).

**Figure 5:**
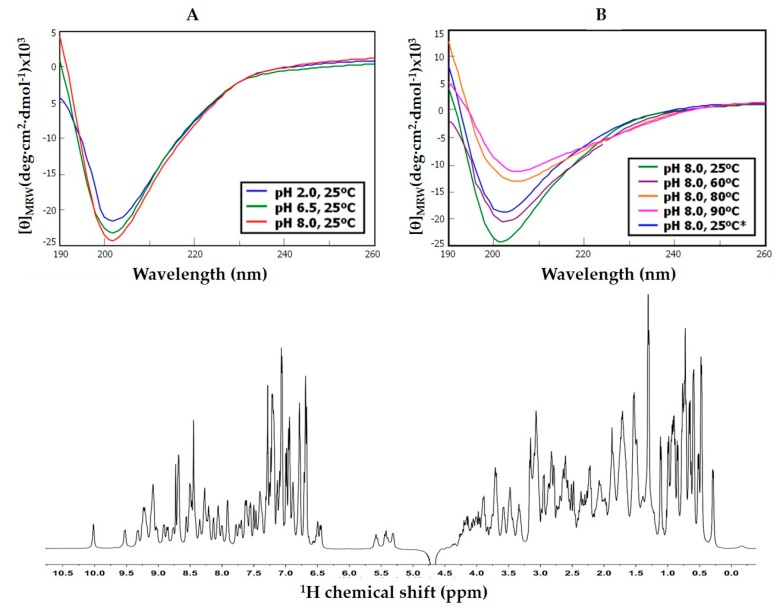
Top: Spectroscopic properties of recombinant NvCI. Top: (**A**) CD spectra at different pH values. (**B**) Thermal denaturation of rNvCI followed by CD. * Indicates the initial temperature reached again after the heat treatment. Bottom: Monodimensional ^1^H 500 MHz NMR spectrum of NvCI at 25 °C.

Both the natural and the recombinant forms of the inhibitor (rNvCI) were analyzed by circular dichroism in the UV region and proton nuclear magnetic resonance spectroscopy at different pH (from 2 to 10) and temperature (from 25 °C to 90 °C) conditions. From the CD spectra, the two forms of the inhibitor evidence a well-folded nature as well as a great stability and capacity to recover its spectral properties when returned to room conditions, with better defined spectra for the recombinant form (Figure 5). Such recovery seems partially compromised when the thermal perturbation is made at pH values above neutrality (as pH 8.0, Figure 5B), probably because of disulphide scrambling. However, refolding is much cleaner when performed at low pH (2.0–6.5) or when the protein is submitted to cysteine reduction at pH 8.0 and heat denaturation (at 90 °C) and, subsequently, allowed to refold for several hours in the presence of redox pairs. Analysis of the refolded form by RP-HPLC, along days at intermediate pH, indicated its homogeneity and practical absence of scrambled forms in equilibrium with the native form, a proof of the high stability of the native state. On the other hand, 500 MHz NMR spectra are very clean and defined, with upfield-shifted methyl resonances indicative of compact-globular state (Figure 5 and Figure S5) and their amide regions appearing completely devoid of alternative or minority resonances, indicating the homogeneity and the correct folding of the sample (see Figure S5 for an example of a bidimensional NMR spectrum). From these observations, it appears that the recombinant form of NvCI is adequate for further studies as, besides its large supply, it allows for a better stability and homogeneity as compared to the natural form (see further on). 

### 2.4. Inhibitory Activity and Selectivity

**Figure 6:**
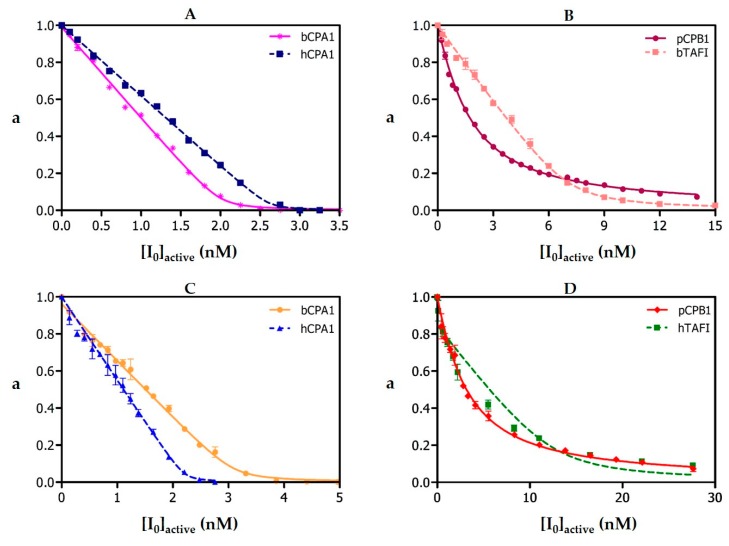
Derivation of equilibrium dissociation constants of NvCI against various metalloproteases. (**A**) Natural NvCI: [bCPA1] = 3.1 nM, [hCPA1] = 2.2 nM. (**B**) natural NvCI: [pCPB] = 4.1 nM, [hTAFI] = 12.5 nM. (**C**) Recombinant NvCI: [bCPA1] = 2.0 nM, [hCPA1] = 2.6 nM. (**D**) Recombinant NvCI: [pCPB] = 1.0 nM, [bTAFI]=7.0 nM. Substrate: 100 mM AAFP for CPA-like enzymes and 100 mM AAFA for CPB-like enzymes. Preincubation time: 15 min, with exception of bTAFI and hTAFI, where the enzyme-inhibitor mixture was not preincubated. Data are means (*n* = 3) ± S.D.

Purified NvCI, either from the natural source or recombinant, is able to strongly inhibit distinct metallocarboxypeptidases (MCPs), particularly those of the M14A subfamily, showing preference for the A1 and A4 variants over A2 and B (Table 1 and Figure 6). In the most outstanding cases, its *K*_i_ values are three orders of magnitude lower than those corresponding to other powerful protein CP inhibitors. In contrast, it does not display substantial inhibitory capability towards the members of the M14B subfamily, such as CPD and CPZ (See Table 1; Table 2). In addition, NvCI is unable to inhibit serine, cysteine and aspartic proteases, even at tenfold excess. The inhibition constants of NvCI against different MCPs were derived using the tight-binding strategy [19,20] from the corresponding titration curves, although the apparent binding kinetics were non-homogeneous: i.e., preincubation of NvCI with different MCPs over different periods of time, in both titration and equilibrium conditions, did not affect the inhibitory effect, indicating a fast binding. An exception was observed for CPA1 forms that showed a slow binding behavior, probably because of their particularly extreme affinity.

It is interesting to note that the natural inhibitor purified from the aqueous extract of the snail displayed about half of its maximum potential activity (50.4%), as shown by competing assays with a similar tight-binding inhibitor as TCI. In contrast, activity approached 95–100% in the case of the inhibitor produced recombinantly. Interestingly, the titration curves displayed a concave shape in several cases (Figure 6), indicating a reversible inhibition, which was mitigated when binding was stronger (for bCPA and hCPA1). On the other hand, analyses of the effect of increasing substrate concentration over inhibitory capability in all the metallocarboxypeptidases tested did not reveal any induced competitive dissociation of the inhibitor, except for hCPA2, where an increase of residual enzymatic activity was observed at substrate concentration over 150 μM, equivalent to 1.5 *K*_M_ (data not shown). Noteworthy is also the observation that the inhibitory capability of the different natural isoforms of NvCI, a, b, c and d, are practically the same, with derived *K*_i_ (nM) of 0.027 ± 0.002, 0.038 ± 0.002, 0.049 ± 0.002, and 0.022 ± 0.001, respectively towards bCPA, an additional evidence of its very high similarity. These are apparent Ki values, without correction by competing assays.

The *K*_i_ values displayed by NvCI against metalloCPs of the M14A family lie within three distinguishable ranges (Table 1). For enzymes such as bCPA, hCPA1 and hCPA4, the very low *K*_i_ values are in the picomolar range; for most of the subfamily members with a different enzymatic activity, i.e., CPB-type enzymes, they are in the order of 1 × 10^−10^ M; and a particular case stands for rCPA3 and for hCPA2 for which NvCI displays a *K*_i_ almost two or four orders of magnitude weaker than for hCPA1, respectively. This behavior allows to conclude that the evolution of inhibitors such as NvCI that act through the insertion of a terminal tail into the enzyme active site reflects both a pressure to fit with the specificity of the enzymes and a pressure to accommodate to the steric constraints posed by the active site architectures. In this regard, it is important to note that the main difference between CPA1 and CPA2 forms does not arise from their preference for the amino acid type but for their volume, as CPA2 prefers bulkier hydrophobic amino acids while CPA1 prefers smaller ones [23]. Overall, however, NvCI represents the tightest-binding carboxypeptidase inhibitor of proteinaceous nature described until now against the M14A protease family.

### 2.5. In Vivo Localization of NvCI

**Figure 7:**
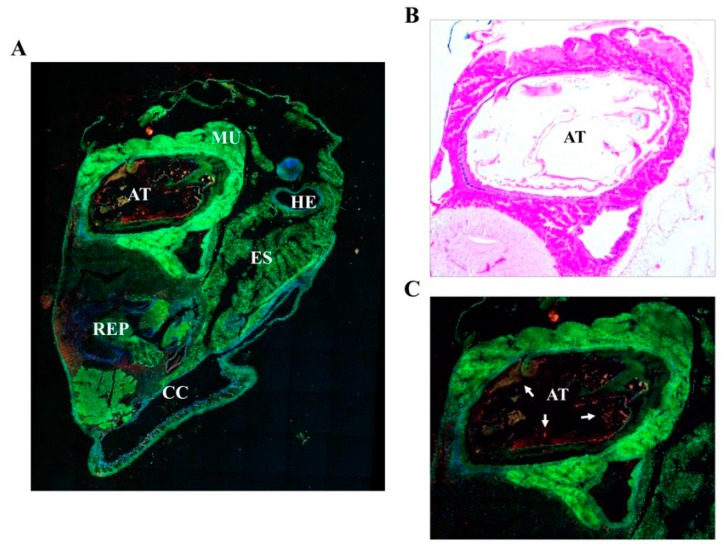
Immunofluorescence localization of NvCI in *Nerita versicolor*. (**A**) Confocal immunofluorescence analysis of the NvCI expression in the posterior region of *N. versicolor.* NvCI (red) was detected using a specific anti-NvCI antibody. Nuclei (blue) were stained with DAPI. Tissue fluorescence is shown in green. (**B**) and (**C**) Detailed view of the alimentary cavity in *N. versicolor* at the posterior region. (**B**), histochemical section and (**C**), Confocal analysis). Abbreviations: AT, alimentary tract; MU, musculature; REP, reproductive tissue; HE, hemolymphatic vessel; CC, celomic cavity; ES, excretory system. The white arrows in C indicate areas with a high NvCI signal.

Several ecological, functional, anatomical and evolutive studies have been conducted on species of the *Neritidae* (*Nerita*) family of snails [24,25,26,27,28,29]. However, in the absence of precise histological anatomical features of *N. versicolor*, we investigated the endogenous expression and localization of NvCI in the snail. Among gastropods, the family *Neritidae* comprises a large number of species capable to live in tropical and subtropical regions and under a wide variety of environmental conditions. While an important number of *Nerita* species are found in freshwater, the most primitive snails live on marine and intertidal rocky ecosystems [24], as is the case of *N. versicolor*. This marine snail, described for first time by Gmelin in 1791, is extensively found in tropical areas from south Florida to Central and South America [29]. In the present study, adult *N. versicolor* specimens were collected in the sea near La Habana (Cuba). Such specimens were used to prepare histochemical sections and perform immunohistochemical analyses in order to study the localization of NvCI in the snail’s anatomy (Figure 7). Immunofluorescence analyses indicate that the strongest signal for NvCI is recognized in the walls of the alimentary tract, particularly nearby its entrance, followed by weaker signals in the reproductive tissue (Figure 7). This suggests the potential involvement of NvCI in the protection of the snail against possible damaging actions of foreign proteases from the environment, i.e., from small marine organisms that could be captured during the pumping and engulfment of surrounding water by the snail or from organisms to which the snail could attach or contact. A similar localization in the intestine was previously reported for ACI, an endogenous carboxypeptidase inhibitor from the intestinal *Ascaris* parasite [10]. By contrast, a very weak or absent staining was found in other organs and tissues of the snail, such as muscles, mantle, connective tissue or excretory system.

### 2.6. Assays on Cell Culture and Biotechnological Potential

Assays on cell toxicity and penetrability were performed in order to establish whether the powerful inhibitor reported here was amenable to practical applications. The effect of NvCI on cell viability was tested in HepG2 cells. This is a cell type useful for cell toxicity studies [30]. None of the NvCI concentrations assayed exhibited substantial cytotoxicity after 72 h incubation time and no perturbations in cell growth, morphology or death were detected (Figure S6). 

**Figure 8:**
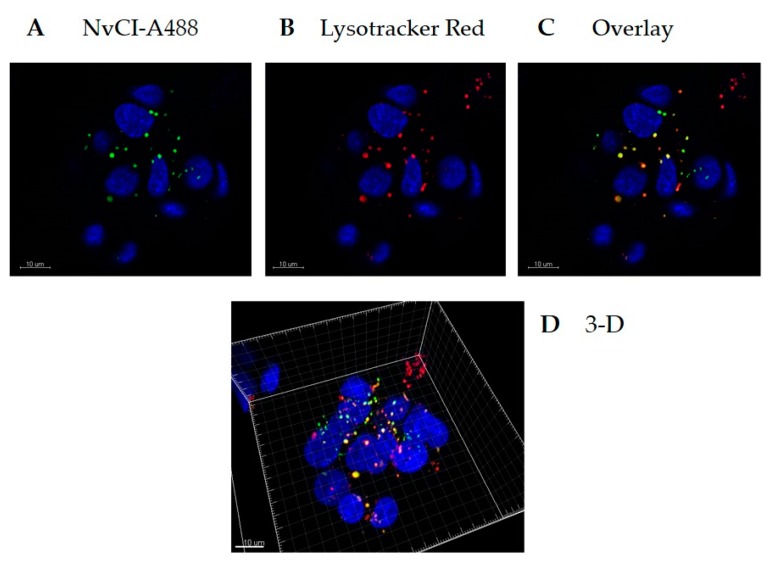
Mammalian cell internalization of NvCI. Live imaging of HepG2 cells incubated 4h with NvCI-Alexa-488 (green) followed by LysoTracker (red) and Hoechst (blue) staining. (**A**) Visualization of the Alexa-488 label. (**B**) Visualization of acidic compartments stained with LysoTracker. (**C**) Overlay of B and C, indicating co-localization of NvCI and LysoTracker. (**D**) 3D reconstruction.

Additionally, we monitored the uptake of labelled NvCI in the same type of HepG2 cells using confocal laser scanning microscopy (CLSM) on Alexa488-labelled NvCI. After 4 h of incubation of NvCI with HepG2 cells, the microscopy analysis demonstrated that internalized NvCI was distributed through the cytoplasm and visualized as smaller accumulations, which appeared to be localized in discrete cellular compartments; co-localization with LysoTracker indicated a distribution in acidic compartments such as lysosomes (Figure 8). In contrast, no fluorescence was detected in untreated (control) cells. The transport mechanism of NvCI through the cell membrane is not known yet, although it may most likely take place through an endocytosis pathway, as previously described for other inhibitors such as cystatins and the carboxypeptidase inhibitor from potatoes [31].

To evaluate the prospects for future biotechnological and biomedical uses of NvCI, we investigated its functional stability in blood and the possibilities for an easy analysis following an affinity-proteomics approach already used by us for complex biological extracts [32]. With such purpose, rNvCI samples at different concentrations were incubated for 1–9 time periods in either human plasma or serum at 37 °C, subsequently recovered by affinity chromatography, and analyzed by HPLC and MALDI-TOF MS. As shown in Figures S7 and S8 the analytical profiles were clean in spite of the very complex plasma or serum environment, facilitating a view of the state of the inhibitor and recovery quantitation. Recovery has been estimated to be around 60–80% in this series (slightly decreasing from 1h to 9h incubation), except at high inhibitor concentration in which it decreased to around 50%, probably due to the saturation of the enzyme in the spin microcolumn, an indication of the requirement of higher loadings of resin. Interestingly, besides the major form at 5945.8 m/z, a minor one (rNvCI-1), at 5798.8 m/z, also appeared from the very beginning and was visualized by HPLC as a minor peak. The difference of around 147.0 m/z corresponds to the N-terminal Phe residue, indicating that it is easily trimmed in both human plasma and serum. The natural protease responsible for the scission is unknown so far. Nevertheless, the trimmed inhibitor keeps functionality as shown by its binding to the affinity resin.

## 3. Discussion

This work reports the characterization of the properties, state and in vivo location of a very compact and stable small proteinaceous inhibitor of metalloCPs in the *Nerita* marine snails that has a powerful inhibitory capacity in the picomolar range, non-previously described for any similar inhibitor of such enzymes.

The purification scheme from natural sources, involving a heating pre-treatment for clarification purposes and two chromatographic steps, is very successful in terms of the massive elimination of unwanted material, part of which was very interfering with purification and also because it allows for the dissociation of the inhibitor from complexes (results not shown) and preserves inhibitory power in better terms than other equivalent strategies used to treat crude extracts, like acid or alkaline precipitation, usage of salts or organic solvents. However, the results obtained from competitive titration indicate that only 50.4% of the isolated natural inhibitor was active, pointing to some structural damage that could be related to certain aspects of the purification treatment, like heating or alkaline release from the affinity column. In contrast, the recombinant production of the inhibitor and its purification by successive fractionation steps using anionic and cationic chromatographies yields pure, active and stable material that, although at the cost of a relatively low purification yield that may be improved in future experiments because of the initial very high expression of the recombinant protein.

The derivation of the amino acid sequence of NvCI, by both direct approach and through cDNA analysis, showed that the protein consists of 53 residues, with a theoretical mass of 5946 Da. The protein has a high percentage of hydrophobic residues (34%, with two Tyr and one Trp), a slightly higher percentage of acidic than basic residues (13% vs. 11%) which equilibrate in the second major isoform, NvCIb, because of the Asn11Lys substitution, and a notable presence of Pro (9%) and Gly (8%). It lacks Met and contains 6 oxidized Cys, expectedly involved in three intrachain disulphide bonds. When the protein and cDNA sequences are aligned (Figure 2; Figure 3), it is evident that the latter has 21 extra nucleotides at the 5′ end, potentially encoding for six additional amino acid residues. In addition, the cDNA sequence of NvCI is also spanning out of the alignment at the 3′ end, after the TGA-stop codon, with 169 untranslated nucleotides plus a polyA tail of 23 residues. The occurrence of additional protein sequences at the N- or C-terminus has been reported for other small protease inhibitors, including those for metalloCPs [10,33], and points to its possible role as pro-sequences potentially involved in initial folding steps.

The availability of a recombinant system to produce large quantities of an active form of NvCI identical or equivalent to the natural one was considered a very important point in this research and for future applications of the inhibitor and has been satisfactorily, but only partially, achieved. The system developed in *Pichia pastoris* cell cultures is still waiting for improvements in the purification scheme but allows producing hundreds of milligrams of highly active inhibitor (rNvCI) in a few days’ time and with reasonable effort. The produced protein is sequentially identical to the natural main form, NvCIa, as shown by proteomic analysis, and displays the same enzymatic parameters (see Table 1), but a higher practical activity by competitive titration (95–98% vs. 50.4% for the purified form from the snail). No evidences of glycosylations or other natural or artifactual chemical residue modifications have been observed.

A key point worth to stress from this work is the unusually high inhibitory power shown by NvCI and its structural-functional implications. The number of proteinaceous small inhibitors of metallocarboxypeptidases characterized in depth so far is small, but it seems to be sufficient to delineate their common characteristics: the members of the main subgroup comprising the smallest ones, have their functionally essential region or primary reactive site at the C-terminus, which is normally 3–5 residues long and docks into the protease active site, as has been reported for inhibitors isolated from potato, tomato, leech, intestinal worms and ticks [5]. On the other hand, the primary interacting site in the largest ones is located in a loop or a region other than the C-terminus, as observed for Latexin [15]. The here characterized NvCI inhibitor clearly fits with the former case, with the difference that the C-terminal tail is even shorter: only two residues. An alignment of the C-terminus of those inhibitors is shown in Figure S9. Since the two terminal residues in NvCI (-Tyr-Ala-COOH) are not substantially different from those in the other aligned positions, the clear improvement in its binding strength to metallocarboxypeptidases (from low-medium nanomolar to pico-subnanomolar *K*_i_, three orders of magnitude) must be related to a much better fit with the enzyme at the primary reactive site and/or to additional effects in the secondary interacting sites. Interestingly, in several previously reported cases, the inhibitor loses one or two C-terminal residues when binding to the protease, after which both become strongly attached, capturing the scissed residue mid-between [7,11] and acting, therefore, as “pseudo-substrates”. This is not the case for NvCI.

Some details of the binding and action mechanism of NvCI on metallocarboxypeptidases started to emerge in a work of our group on the NvCI-hCPA4 complex, previously reported because of the easy crystallization and X-ray analysis of the complex [14], when many characteristics of the whole system were still under preliminary study. In short, it was hypothesized that whilst in the other small proteinaceous inhibitors of metallocarboxypeptidases the primary reactive site only covers the S1 and S2 subsites of the enzyme, in the case of the NvCI-hCPA4 complex the inhibitor also covers the S3 subsite through a double hydrogen bond between residues Cys51 (involved in a disulfide bond) and Tyr52, and residue Glu163 of the enzyme. This would be based on a different orientation of Cys51 which, in turn, would give rise to a richer hydrogen bonding network between NvCI and hCPA4. Another factor could be the larger interface found between both counterparts as compared to other cases. It may seem surprising that such small changes can give rise to three orders of magnitude change in the inhibition constants in the extreme cases. Further experimentation is required for a deeper interpretation. In the present work, we have shown that such extraordinary binding power and low *K*_i_ values previously found for hCPA4 are matched or even beaten in complexes of NvCI with bCPA and hCPA1 in which the Glu163 position is kept. It also suffers a weakening in complexes with enzymes that maintain Glu163 but differ in C-terminal specificity (hCPB and hTAFI) and dramatically decrease in the complex with hCPA2 due to the interplay of two factors: the substitution Glu163Asp and a selectivity pocket designed to host residues bigger than Val. Such advance in knowledge could facilitate its future engineering, i.e., towards more specific or convenient forms.

The main in vivo location of NvCI in the surface and the regions of the animal facing the open entrance of the shell and gut suggests a potential functional role of NvCI in the protection of the snail against possible damaging actions from the environment. In fact, this hypothesis would fit with the increasingly convincing evidences that biological defense and protection against predators or invading species is one of the main properties that explain the occurrence of such protease inhibitors in marine invertebrates [34], as well as in animals and plants, in general [35,36]. Specific activity studies and analysis of the biological niche would be required to substantiate this view for NvCI. It would be interesting to investigate whether this hypothesis can be extended to other marine invertebrates in which the occurrence of proteinaceous inhibitors of M14 MCPs is known. This would be the case of the well based work on the inhibitor in *Sabellastarte magnifica* [16], or the just initially unveiled occurrence of equivalent inhibitors in other eight marine invertebrates encompassing Mollusca, Cnidaria, Annelida and Chordata phyla, recently reported by our group [32].

A remaining question is to what extend NvCI could have biotechnological and/or biomedical potential uses as a drug or lead compound given its exceptional capability to inhibit metallocarboxypeptidases and the growing evidences on the involvement of those enzymes in important biological mechanisms and diseases [5,37]. In favor of such uses are its lack of toxicity when added to cellular assays, its stability in the presence of human blood plasma or serum and the fairly abundant recombinant production of NvCI shown in this work. This indication is also congruent with the frequently reported short-term stability, innocuousness and bioavailability of related proteinaceous inhibitors when tested in cellulo and in vivo in mice and humans [38]. At this respect, it is worth remembering that a well-established circulatory carboxypeptidase, plasma Carboxypeptidase B or TAFIa [39], is a key factor in the stabilization of blood clots and an important target for fibrinolytic therapeutic strategies based on inhibitors [40]. Also, that mastocytosis is a growing conjoint of diseases [41] that gives rise, among others, to massive extracellular release of carboxypeptidase A3 from degranulated mastocytes, constituting a potential therapeutic target for carboxypeptidase inhibitors [42]. Both enzymes are strongly inhibited by NvCI, as shown in Table 1. Overall, the very small, compact and stable NvCI proteinaceous inhibitor, which is potent and specific for the carboxypeptidase M14A subfamily, can be of practical use, either directly, or after redesign towards increased specificity or as minimized forms.

## 4. Materials and Methods 

### 4.1. Purification of Natural NvCI

*Nerita versicolor* snails were collected in the tropical sea near La Habana (Cuba) and validated by the Cuban Oceanographic Institute. The body of the snails were removed from the shell, washed with seawater, homogenized in a home blender, tissue-filtered, clarified by heating at 60 °C for 30min, centrifuged at 6000 rpm, freeze-dried and kept at −20 °C. After solubilization in the column equilibration buffer (500mM NaCl, 20mM Tris-HCl, pH 7.5), the solution was centrifuged again and loaded on a CPA-glyoxal Sepharose^®^ CL-4B column (1.6 × 9.9 cm, containing 3.5mg of CPA/ml gel) [31]. Non-retained molecules were removed by washing the column with equilibration buffer and elution was performed with 10 mM NaOH, pH 12.0. The whole process was carried out at room temperature. The elution profile was followed at 280nm in a Pharmacia FPLC equipment (GE Amersham, UK) and peaks were collected in tubes containing a neutralizing buffer. The fraction containing NvCI was then applied to a Jupiter 0.39 × 15cm HPLC-C4 column (Phenomenex, Torrance, CA, USA), equilibrated and washed with 0.1% TFA in water (solution A), and elution was performed with 0.1% *v/v* TFA in acetonitrile (solution B) using the following gradient: 10% of solution B during 15 min followed by a linear gradient from 10 to 40% over 100 min and a linear gradient from 40 to 98% over 1 min. The flow rate was 0.5 ml/min at room temperature. After evaporation of the organic solvent, the fractions containing NvCI, as verified by MALDI-MS and inhibition analysis, were freeze-dried and kept at −80 °C. Total protein concentration was measured in the crude extracts using the bicinchoninic acid (BCA) method [43].

### 4.2. Primary Structure Determination

The NvCIa isoform was dissolved in 50mM TrisHCl (pH 8.0) and 2% SDS, or 6M guanidinium chloride, heated at 95 °C for 5 min, centrifuged at 6000 rpm for 20 min and the supernatant transferred to a clean tube. The sample was reduced with 20 mM DTT at 56 °C for 30 min, alkylated with iodoacetamide (20 mM, at 25 °C, for 30 min, in the dark), desalted and concentrated by ZipTipC4 pipette tips (Millipore, Burlington, MA, USA). MALDI-TOF-MS analysis using DHAP as a matrix were made before and after reduction-alkylation to derive the number of free- or disulfide-linked cysteines in the molecule. Subsequently, an aliquot of the reduced-alkylated protein was subjected to eighteen cycles of automated EDMAN degradation. Additional aliquots were treated with Lys-C endoproteinase trypsin and Glu-C endoproteinase at a 40/1 w/w ratios and for 1h at 37 °C and the digest were desalted using Zip TipC4 pipette tips and analyzed by MALDI.TOF-MS using α-CHCA as a matrix to generate a peptide mass fingerprint (PMF). The peptide fragments were subsequently subjected to CID fragmentation within the spectrometer, using the LIFT method and experimental details previously reported [32]. Peptide alignment and protein sequence analyses from the fragmentation spectra were performed using the Bruker Daltonics software and Mascot search engine, also following the mentioned previous report, with incorporation of the Edman degradation data and the help of the parallel analysis of the NvCI cDNA. Comparison of the PMF and fragmentation data derived from each NvCI isoform (a, b, c and d) allowed to derive the corresponding sequences and differences.

Total RNA was isolated from the marine snail body using the Nucleospin kit (Macherey-Nagel, Düren, Germany), and poly(A) + RNA was purified using the Nucleotrap kit (Macherey-Nagel, Düren, Germany) both according to manufacturer’s instructions. The first strand of NvCI cDNA was synthesized using the adaptor oligonucleotide R_0_R_1_polydT (5´CCGGAATTCACTGCAGGGTACCCAATACGACTCACTATAGGGCTTTTTTTTTTTTTTTTT-3´) and avian myeloblastosis virus reverse transcriptase according to the supplier’s protocols. For cloning the NvCI cDNA, four semi-degenerated oligonucleotides were designed based on its N-terminal sequence, P1_NvCI_1-8_: 5’-TTYCAYGTSCCNGAYGAYCGNCC-3’, P2_NvCI_1-8_: 5’-TTYCAYGTSCCNGAYGAYAGRCC-3’, P3_NvCI_1-8_: 5’-TTYCAYGTWCCNGAYGAYCGNCC-3’ and P4_NvCI_1-8_: 5’- TTYCAYGTWCCNGAYGAYAGRCC -3’. Where, Y = C/T, R = A/G, S = C/G, W = A/T, N = A/C/G/T.

In order to isolate the specific gene product of the RT-PCR, a PCR step was subsequently performed using the specific primers for NvCI and the R_0_ primer: 5´-CCGGAATTCACTGCAGGGT-3´. PCR was conducted as 35 cycles each at 95 °C for 30s, annealing at 60 °C for 30 s, and extension at 72 °C for 90 s. PCR products were separated by electrophoresis on 2% agarose gels. Selected and purified PCR product was cloned into pBE vector to generate the pBE-NvCI construct (corresponding to NvCI amino acid residues from 1 to 53).

### 4.3. Recombinant Production and Biophysical Analysis of rNvCI

#### 4.3.1. Cloning, Expression and Purification in Pichia Pastoris

The plasmid construct for recombinant NvCI was based on a synthetic codon-optimized sequence (Geneart, Regensburg, GE), fused in frame to the *Saccharomyces cerevisiae* prepro α-factor signal under the AOX1 gene promoter. Zeocin hyper-resistant *P. pastoris* transformants (at high antibiotic concentration) were selected to generate enrichment in recombinant strains with multiple copies of the integrated vector. Recombinant production of NvCI was carried out in a 3 L autoclavable bioreactor (Applikon Biotechnology B.V., Delft, Netherlands), methanol autocontrolled, monitored by cell density, cell weight, protein concentration (by the BCA method) as well as NvCI concentration and integrity (by HPLC, MALDI-MS and activity) along the three and a half days of operation (36 h cell growth until 129g/L cell wet weight, followed by 48 h of induction by 2–3 g methanol per liter of culture, at 25 °C and pH 4.0. According to protein and NvCI analyses, 550 mg/L of recombinant NvCI were obtained in the centrifuged culture broth and about 330 mg/L were produced as a final purification yield at the end of the process.

The purification scheme comprised two steps: (1) A weak cation exchange chromatography of the rNvCI fermentation supernatant on a Waters Accell™ Plus CM column (1.6 cm × 20.0 cm) using buffers A (20 mM sodium citrate, pH 3.0), B (20 mM Tris-HCl pH 7.0) and C (20 mM Tris-HCl pH 7.0, containing 1 M NaCl). The column was equilibrated at 1 ml/min with buffer A over 3 column volumes (CV). Sample loading was performed at 1 ml/min in buffer A. Non-retained molecules were removed by washing the column at 1 ml/min with buffer A and 3 CV. Elution was performed starting with 100% buffer B, at 2 ml/min and 4 CV, followed by a linear gradient from 0 to 100% C at 2 ml/min with 4 CV, and 100% C at 2 ml/min and 1 CV. The whole process was carried out at room temperature. (2) A weak anion exchange chromatography of the elution peak from the previous chromatography on a TSK-GEL™DEAE-5PW column (7.5 cm × 7.5 mm) using buffers A (20 mM Tris-HCl pH 8.5) and B (B: 20 mM Tris-HCl pH 8.5, containing 1 M NaCl). The column was equilibrated with buffer A over 5 CV. Sample loading was performed in buffer A. Non-retained molecules were removed by washing the column with buffer A over 5 CV. Elution was performed using a linear gradient from 0% to 100% B over 20 CV followed by 100% B over 10 CV. The flow rate was 1 ml/min at room temperature. The purified sample was analyzed by MALDI-TOF MS on a MTP 384 target plate polished steel T F (Bruker Daltonics), followed by deposition of 1 μL of DHAP as a matrix. The purified sample was subsequently reduced and S-carbamidomethylated using the procedures described in [32]. 

#### 4.3.2. Biophysical Analyses

The folding state and stability of NvCI was investigated on the natural and recombinant forms by CD. The proteins, either in 0.1% TFA (v/v) or 20mM sodium phosphate (pH 2.0, 6.5 or 8.0) were analyzed at 0.05–0.06 mg/ml, from 25 to 90 °C in a Jasco J-710 spectropolarimeter (Pfugnstadt, Germany) in the 190–320nm range. Additional analyses were made under folding conditions (20mM sodium phosphate, pH 8.0) in the absence and presence of reducing reagent (1 or 20 mM DTT), a redox-pair (1mM Cys + 0.5mM cystine), and either absence or presence of denaturant (8 M urea). Complementary ^1^H-NMR experiments were performed in a 500MHz NMR (Bruker Advance) on rNvCI in 20 mM sodium phosphate (pH 2.0 and 6.5), in 90% ^1^H_2_O + 10% D_2_O, at 298 K. DQF-COSY, NOESY, TOCSY and HSQC spectra were obtained in a TCI cryoprobe of 5mm and used for provisional backbone assignment. 

### 4.4. Enzymatic Analysis of the Inhibitory Activity

Inhibitory activities against CPA- and CPB-type forms (bovine and human variants of CPA1, CPA2, CPA4, CPB1, and TAFI) were assayed for all extracts, subject to the experimental conditions that enzyme (E), substrate (S) and inhibitor (I) concentrations as well as the [I]/[E] ratio and preincubation time followed the requirements for tight-binding inhibitor assays [44]. Similar approaches were followed to measure the activities of proteases of different catalytic types, like pepsin, papain, trypsin and subtilisin [45,46,47,48]. All assays were performed in triplicate at 25 °C in a multiplexed manner. For 96-well assays an iEMS spectrophotometer reader/dispenser FM (Labsystems, Finland) was used with a final reaction volume of 250 μL. For cuvette assays, a Cary 400 Bio spectrophotometer (Varian Inc., Palo Alto, CA, USA) spectrophotometer was used with a final reaction volume of 1 ml. The reactions were followed at 5 s intervals for 5 min and recorded as initial velocities. In the protease inhibition assays, mixtures of activity buffer, biological sample and enzyme were preincubated at 25 °C for 10min, before substrate addition. The assay conditions were as follows: for CPA-like enzymes, 7.0nM CPA, 0.1mM AAFP substrate in 20mM Tris-HCl pH 7.5, 500mMNaCl, 1% *v/v* dimethyl sulfoxide (DMSO), 0.05% *w/v* BRIJ-35 was used [49]. For CPB-like enzymes, CPB 3.0nM CPB, 0.1mM AAFA substrate and activity buffer of 20 mM Tris-HCl pH [50].

The *K*_i_ values of natural and recombinant NvCI against bCPA1, hCPA1, hCPA2, rat CPA3, hCPA4, hCPB1, bTAFI, hTAFI, hCPD and hCPZ were determined by measuring the enzymatic residual activity (*V*_i_/*V*_0_ = a) at different inhibitor concentrations and using a fixed enzyme and substrate concentrations as described above, where v_i_ and v_0_ are the fraction of enzymatic activity in the presence and absence of inhibitor, respectively, in terms of initial velocities. The determination of *K*_i_ values was carried out on equilibrium conditions ([E_0_]/Ki ≤ 10), using a previously determined preincubation time of 15 min, required to establish enzyme-inhibitor equilibrium. Working temperature was 37 °C. The best estimates of *K*_i_ values were obtained by fitting the experimental data to the equation for tight-binding inhibitors [19] by non-linear regression using the GraphPad Prisma 5 software (GraphPad Software, Inc., San Diego, CA, USA) at *p* < 0.05. The real *K*_i_ values against each of these A/B-type MCPs were calculated according to the equation described by Morrison and Copeland [19,20].

### 4.5. Histology and Immunofluorescence Experiments

Adult *Nerita versicolor* specimens were obtained from the coast nearby La Habana, Cuba, and left several days only in sea water and without food intake. Whole bodies were extracted from the shell and operculum and fixed in 10% formalin, embedded in paraffin, and cut into 5-µm serial sections. A set of histological sections were stained with hematoxylin and eosin following standard protocols. For immunolocalization experiments, sections were stained with fluorescent-labelled antibodies raised against rNvCI. Such antibodies were produced in house by the SCAC (Servei de Cultius Cel·lulars, producció d´Anticossos i Citometria) at the UAB. The antibody did not cross react with other proteins presents in the *Nerita versicolor* extract, as tested by Western blot analysis. All incubations were performed at room temperature in a humid chamber. After deparaffinization and rehydration, the sections were immersed in 3% H_2_O_2_ in distilled water for 35 min to block endogenous peroxidase activity and then washed three times with distilled H_2_O. The slides were placed in 10 mM sodium citrate, pH 6.0, and incubated in a water bath at 98 °C for 20 min. Next, the preparations were washed 3 times with PBS, pH 7.4, and blocked with normal goat serum (30%) in PBS for 1 h at room temperature. The sections were then incubated overnight with primary antibody (dilution 1:50) in normal goat serum (30%) in PBS for 1 h at room temperature. The sections were then incubated overnight with primary antibody (dilution 1:50) in normal goat serum (30%) in PBS at 4 °C. After incubation, sections were washed three time with buffer and incubated with an Alexa647 labelled secondary antibody (goat anti-rabbit antibody, dilution 1:500) for 30 min. After washing, sections were incubated with DAPI for 15 min at room temperature and washed with PBS buffer. Finally, the sections were mounted with Prolong Gold mounting medium (Molecular Probes, Eugene, OR, USA). As a negative control, preimmune serum instead of a primary antibody was used. Fluorescence imaging was acquired in a Leica TCS SP5 (Wetzlar, Germany) confocal fluorescence microscope.

### 4.6. Cytotoxicity Evaluation of NvCI

Cytotoxicity effect of NvCI was tested in a cell culture system using the human hepatocyte cell line HepG2 (American Type Culture Collection (ATCC)). The cells were grown in Dulbecco’s modified Eagle’s medium (DMEM) medium supplemented with 10% (v/v) heat inactivated fetal bovine serum, 2 mM glutamine (Life Technologies Inc.), in a highly humidified atmosphere of 90% air with 10% CO_2_ at 37 °C. Growth inhibitory effect was measured by the microculture tetrazolium [2,3-bis-(2-methoxy-4-nitro-5-sulfophenyl)-2H-tetrazolium-5-carboxanilide, XTT] assay [51]. Following the addition of different inhibitor concentrations to quadruplicate wells, plates were incubated at 37 °C for 72 h. Aliquots of 20 µl of XTT solution were then added to each well. After 3 h, the color formed was quantitated by a spectrophotometric plate reader (PerkinElmer Victor3 V) at a wavelength of 490 nm. Cell viability was expressed as a percentage of the control level. 

### 4.7. Cellular Uptake of NvCI

Recombinant NvCI was fluorescently labelled with an Alexa Fluor 488 Protein Labeling Kit (Molecular Probes, Eugene, OR, USA). HepG2 cells were seeded in a 35 mm glass-bottom culture dish (MatTek Corporation, Ashland, MA, USA) at a density of 4 × 10^5^ cells/well and incubated for 24 h. The cells were incubated with 20 µg/ml Alexa 488-NvCI for 4 h at 37 °C. Prior to imaging, lysosomes and nuclei were stained with Lysotracker Red and Hoechst (Invitrogen, Carlsbard, CA, USA), respectively, for 90 or 10 min. 

### 4.8. Molecular and Functional Stability of PCI in Blood Plasma and Serum

rNvCI was dissolved in human plasma and serum samples (provided by Sigma-Aldrich, Saint Louis, MO, USA), at concentrations ranging from 0.001 to 0.5 mg/mL, the mixture was incubated at 37 °C, 100 μL aliquots were withdrawn at 1, 3 and 9 h, and mixed with 100 μL of 40 mM Tris-HCl, 1MNaCl, pH 7.5. The aliquots were analyzed by affinity proteomics on human CPA-microaffinity spin columns (100 μL each, from ThermoFisher, Waltham, MA, USA), followed by RP-HPLC on a C4 column and MALDI-TOF.MS, by a procedure and conditions for biological extracts described previously [32]. The captured NvCI was released by acid treatment with 110 μL of 0.5% v/v TFA, and 95 μL were applied on a RP Jupiter 5u C4 300A 250 × 10 mm (Phenomenex) HPLC column, with detection at 214 and 280 nm. A calibration graph for NvCI was derived in the 0.001-0.5 range, as shown in Fig.S7. The remaining 10 μL were desalted in ZipTip C4 (Millipore, Burlington, MA, USA) and analyzed by MALDI-TOF MS in a Bruker Xtreme (Billerica, MA, USA) spectrometer.

## Figures and Tables

**Table 1:** Summary of *K_i_* values of natural and recombinant NvCI against various carboxypeptidases.

Enzyme	NvCI *K_i_* (pM)	rNvCI *K_i_* (pM)
bCPA1	9.6 ± 1.4	5.8 ± 0.2 (a)
hCPA1	1.8 ± 0.2	1.2 ± 0.1 (a)
hCPA2	6507.3 ± 68.6	2941.0 ± 132.4 (a)
rat CPA3	-	100.3 ± 20.7
hCPA4	9.7 ± 1.0	4.9 ± 0.6 (a)
pCPB	649.9 ± 12.2	334.7 ± 4.5
hCPB	110.9 ± 4.7	549.3 ± 2.4
bTAFI	121.4 ± 20.1	84.1 ± 13.4
hTAFI	392.9 ± 21.9	950.9 ± 98.8
hCPD	NI	NI
hCPZ	NI	NI

Data are means (*n* = 3) ± SD. *K*_i_ in nM. NI: No inhibitory activity detected for the [I] 0 range from 1 nM to 1 mM. **a**. Values taken from Covaleda et al., 2012 [14].

**Table 2:** *K_i_* values of various proteinaceous metallocarboxypeptidases (MCPs) inhibitors.

Carboxypeptidase	*K_i_* (nM)				
	rPCI (a)	rTCI (b)	rLCI (c)	rACI (d)	rhuman latexin (e)
bCPA1	1.6 ± 0.2	1.1 ± 0.3	1.1 ± 0.2	2.4 ± 0.3	1.2 ± 0.2
hCPA1	1.6 ± 0.3	1.2 ± 0.4	1.3 ± 0.4	1.6 ± 0.2	1.6 ± 0.2
hCPA2	8.8 ± 0.7	3.6 ± 0.5	4.5 ± 0.6	2.5 ± 0.2	3.5 ± 0.3
hCPA4	1.3 ± 0.1 (f)	0.8 ± 0.3 (f)	7.3 ± 0.4 (f)	23.9 ± 3.1	3.0 ± 0.3
pCPB	7.2 ± 0.6	1.6 ± 0.3	2.4 ± 0.5	NA	NA
hCPB	1.8 ± 0.3	1.3 ± 0.2	1.2 ± 0.3	NA	1.1 ± 0.1
bTAFI	2.5 ± 0.4	1.3 ± 0.3	NA	NA	NA
hTAFI	5.2 ± 0.5	1.2 ± 0.4	NA	42.0 ± 1.7	1.8 ± 0.3

**a, b**. *K*_i_ values from Arolas et al., 2005 [12]. **c**. *K*_i_ values from Arolas et al., 2004 [21]. **d**. *K*_i_ values from Sanglas et al., 2009 [10]. **e**. *K*_i_ values from Pallarès et al., 2005 [15]. **f**. *K*_i_ values from Tanco et al., 2010 [22]. NA: data not available.

## Supplementary Materials

The following are available online at https://www.mdpi.com/1660-3397/17/9/511/s1, Figure S1: MALDI-TOF spectra of subfractions NvCIa, NcCIb, NvCIc and NvCId; Figure S2: MALDI MS/MS spectrum of parent m/z = 2980 from NvCI enzymatic digestion with Lys-C; Figure S3: Analysis of NvCI isoforms by peptide mass fingerprinting (PMF); Figure S4: Molecular characterization of rNvCI by MALDI-TOF; Figure S5: ^1^H 500MHz 2D-NMR spectra of NvCI; Figure S6: Effects of NvCI on cytotoxicity/cell viability; Figure S7: Calibration plot of the analytical quantitation of rNvCI incubated in blood plasma or serum, after spin-micro affinity capture followed by fraction recovery and HPLC analysis; Figure S8: Analysis of homogeneity and state of rNvCI by HPLC (top) and MALDI-TOF MS (bottom), after incubation and recovery from blood plasma and serum; Figure S9: Comparison of the C-terminal amino acid sequences of NvCI and other proteinaceous MCPs inhibitors.
