# Supplementary material for: Characterization, Recombinant Production and Structure-Function Analysis of NvCI, A Picomolar Metallocarboxypeptidase Inhibitor from the Marine Snail Nerita versicolor
Source: Mar Drugs. 2019 Aug 29;17(9):511. doi: 10.3390/md17090511 (PMC6780499; doi:10.3390/md17090511)
Supplement: Supplementary file 1 [file marinedrugs-17-00511-s001.zip › GCovaleda.MD-SupplemFile&HighResolFigs.25Aug/GCovaleda_CharacterizationNvCI_MD.SupplemMat.2019VIII25.F.docx]

Supplementary Material for

Characterization, Recombinant Production and Structure-Function Analysis of NvCI, a Picomolar Metallocarboxypeptidase Inhibitor from the Marine Snail *Nerita versicolor*

Giovanni Covaleda-Cortes^1,§^, Martha Hernández^2,§^, Sebastián Alejandro Trejo^1^, Manuel Mansur^1^, Sergi Rodríguez-Calado^1^, Javier García-Pardo^1^, Julia Lorenzo^1, *^, Josep Vendrell^1^, María Ángeles Chávez^3^, Maday Alonso del Rivero^3,*^ and Francesc Xavier Avilés^1,*^

^1^ Institut de Biotecnologia i de Biomedicina and Departament de Bioquímica, Universitat Autònoma de Barcelona, 08193 Bellaterra (Barcelona), Spain; [gcovaledacortes12@gmail.com](mailto:gcortes12@gmail.com) (G.C.); [sebatrejo@gmail.com](mailto:sebatrejo@gmail.com) (S.A.T.); [m_2906@yahoo.es](mailto:m_2906@yahoo.es) (M.M); [sergi.rodriguez@uab.cat](mailto:sergi.rodriguez@uab.cat) (S.R-C.); [javier.garciap@uab.cat](file:///E:\Recerca\Treballs%202012-\NvCI\javier.garciap@uab.cat) (J.G-P.) [Julia.Lorenzo@uab.cat](mailto:Julia.Lorenzo@uab.cat) (J.L.); [Josep.Vendrell@uab.cat](mailto:Josep.Vendrell@uab.cat) (J.V.); [FrancescXavier.Aviles@uab.es](mailto:FrancescXavier.Aviles@uab.es) (F.X.A.)

^2^ Facultad de Ciencias Forestales. Centro de Biotecnología. Universidad de Concepción, Victoria 631, Barrio Universitario, Concepción, Chile; [marhernandez@udec.cl](mailto:marhernandez@udec.cl) (M.H.)

^3^ Centro de Estudio de Proteínas, Facultad de Biología, Universidad de la Habana, La Habana, Cuba; [maday@fbio.uh.cu](file:///E:\Recerca\Treballs%202012-\NvCI\maday@fbio.uh.cu) (M.A.R.); [machavez1229r@gmail.com](mailto:machavez1229r@gmail.com) (M.A.C.)

^§^ Both authors have contributed equally to this work

***** Correspondence: [FrancescXavier.Aviles@uab.es](mailto:FrancescXavier.Aviles@uab.es) (F.X.A.); [maday@fbio.uh.cu](mailto:maday@fbio.uh.cu) (M.A.R.); [Julia.Lorenzo@uab.cat](mailto:Julia.Lorenzo@uab.cat) (J.L.)

[Figure S1. MALDI-TOF spectra of subfractions NvCIa, NcCIb, NvCIc and NvCId (A, B, C, D, respectively) 2](#_Toc15558879)

[Figure S2. MALDI MS/MS spectrum of parent m/z=2980 from NvCI enzymatic digestion with Lys-C. 3](#_Toc15558880)

[Figure S3. Analysis of NvCI isoforms by peptide mass fingerprinting (PMF). 4](#_Toc15558881)

[Figure S4. Molecular characterization of rNvCI by MALDI-TOF. 5](#_Toc15558882)

[Figure S5. ^1^H 500MHz 2D-NMR spectra of NvCI. 6](#_Toc15558883)

[Figure S6. Effects of NvCI on cytotoxicity/cell viability. 7](#_Toc15558884)

[Figure S7. Calibration plot of the analytical quantitation of rNvCI incubated in blood plasma or serum, after spin-micro affinity capture followed by fraction recovery and HPLC analysis. 8](#_Toc15558885)

[Figure S8. Analysis of homogeneity and state of rNvCI by HPLC (top) and MALDI-TOF MS (bottom), after incubation and recovery from blood plasma and serum. 9](#_Toc15558886)

[Figure S9. Comparison of the C-terminal amino acid sequences of NvCI and other proteinaceous MCPs inhibitors. 11](#_Toc15558887)


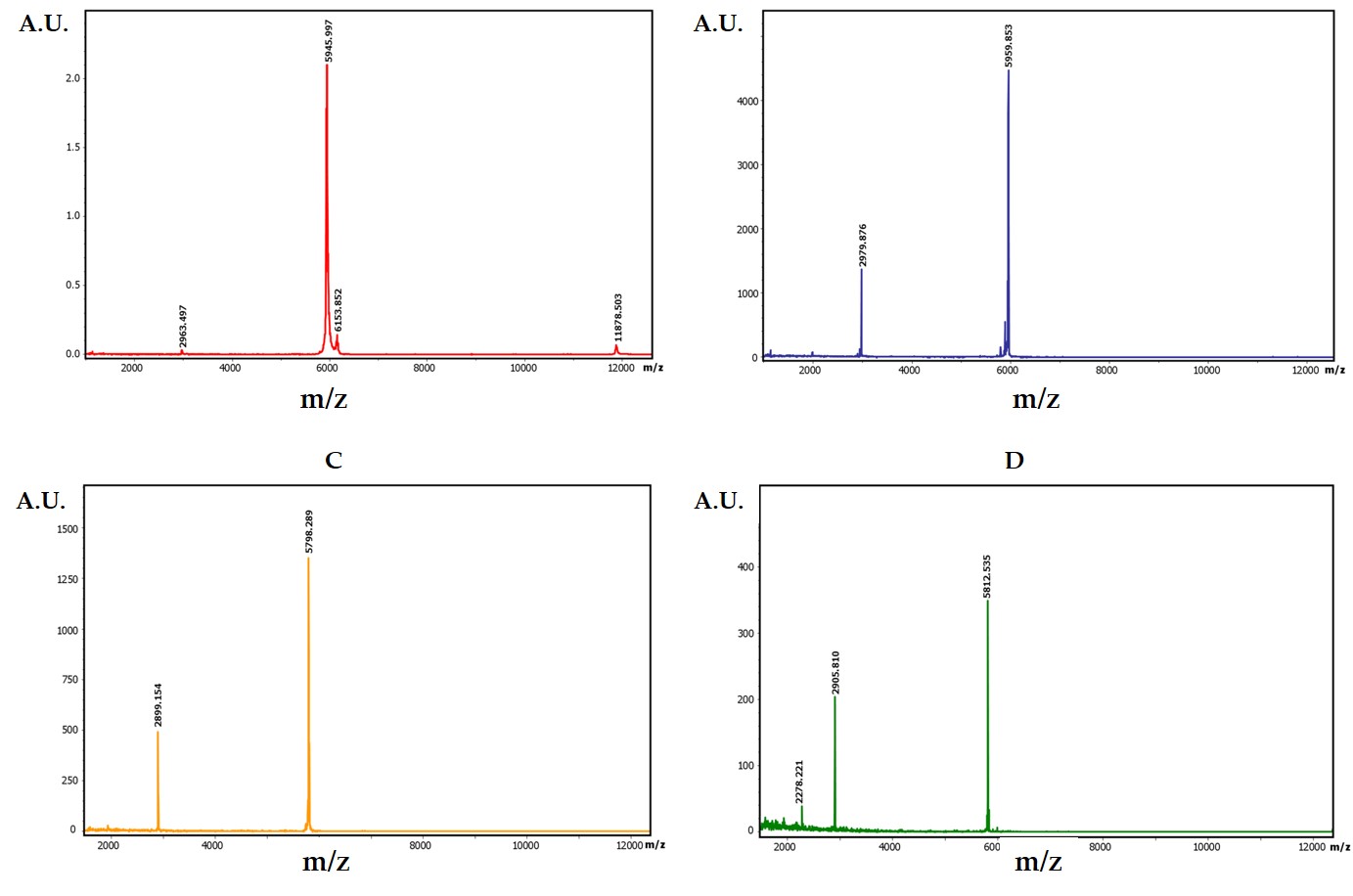
**Figure S1. MALDI-TOF spectra of subfractions NvCIa, NcCIb, NvCIc and NvCId (A, B, C, D, respectively**)


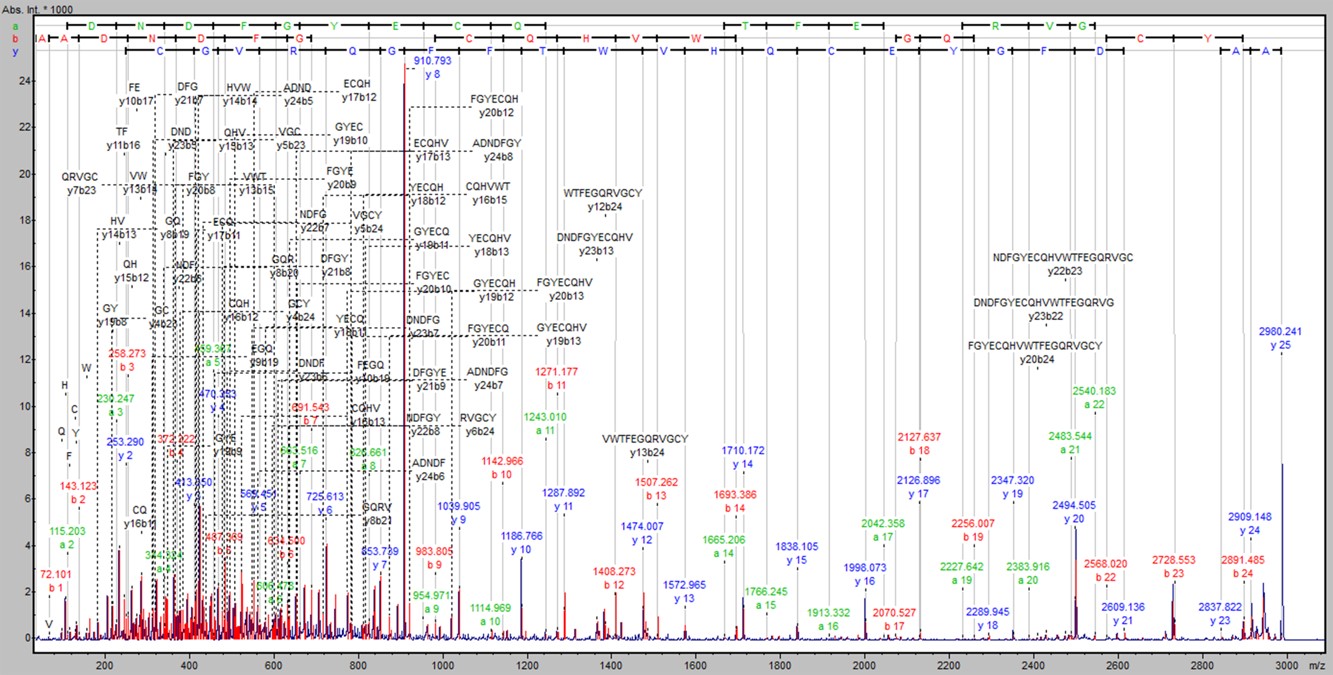


# Figure S2. MALDI MS/MS spectrum of parent m/z=2980 from NvCI enzymatic digestion with Lys-C.


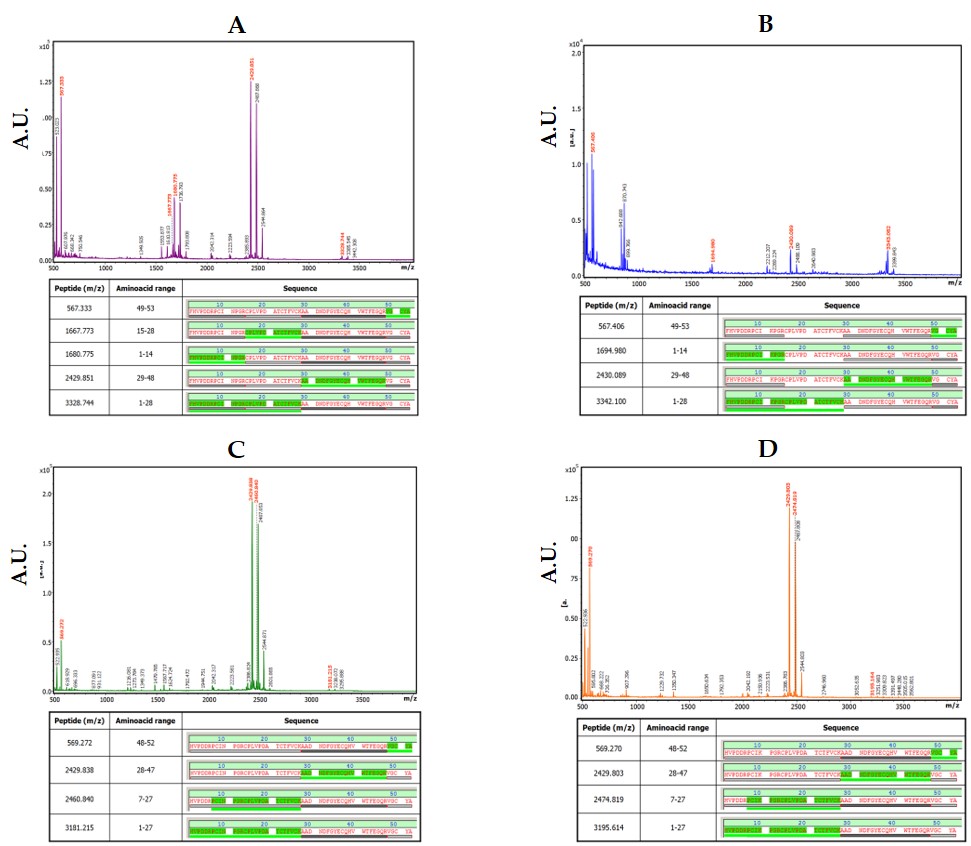


# Figure S3. Analysis of NvCI isoforms by peptide mass fingerprinting (PMF).

MALDI spectra of the tryptic digests of NvCI (A), NvCIb (B), NvCIc (C) and NvCId (D)


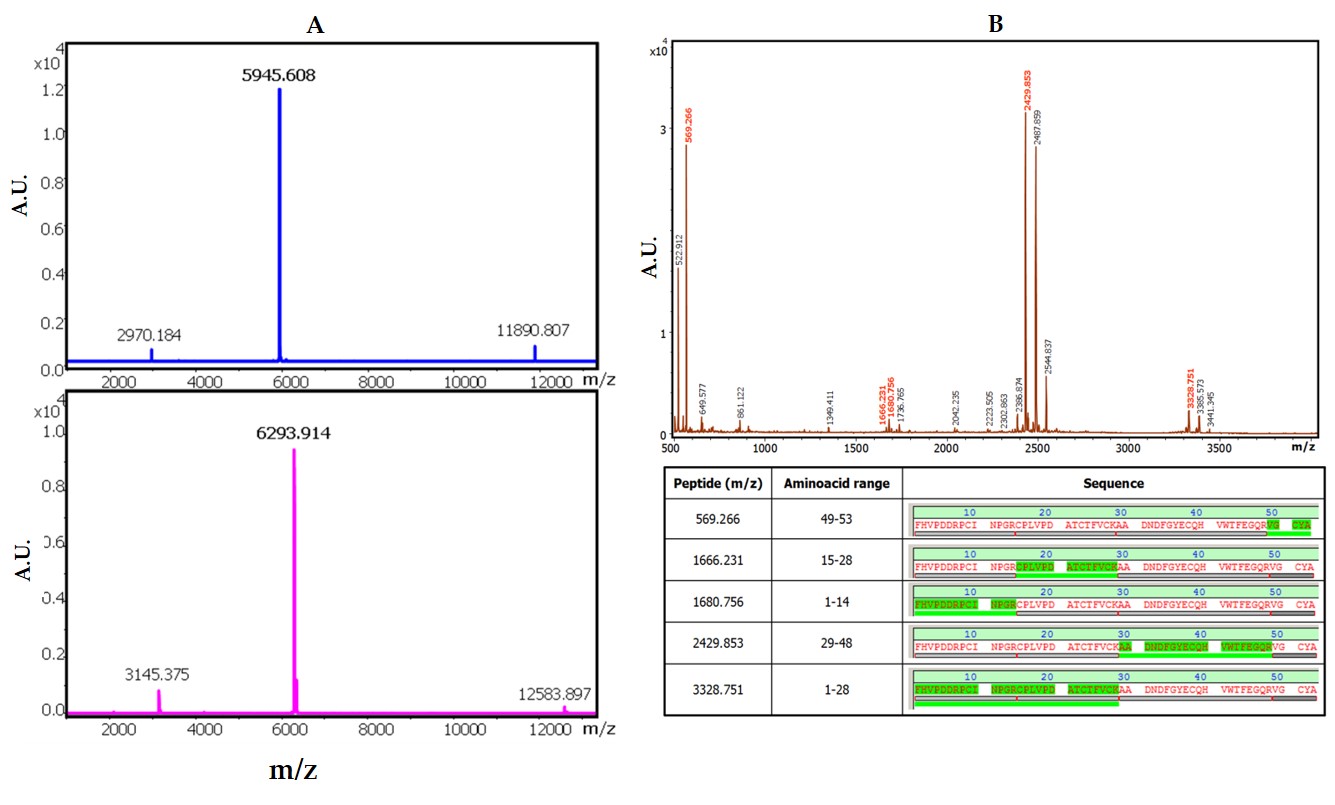
**Figure S4. Molecular characterization of rNvCI by MALDI-TOF**.

A. Upper: MALDI-TOF MS spectrum of rNvCI; lower: MALDI-TOF MS spectrum after reduction and S-carbamidomethylation. B. Peptide mass fingerprint of the tryptic digestion of NvCI.


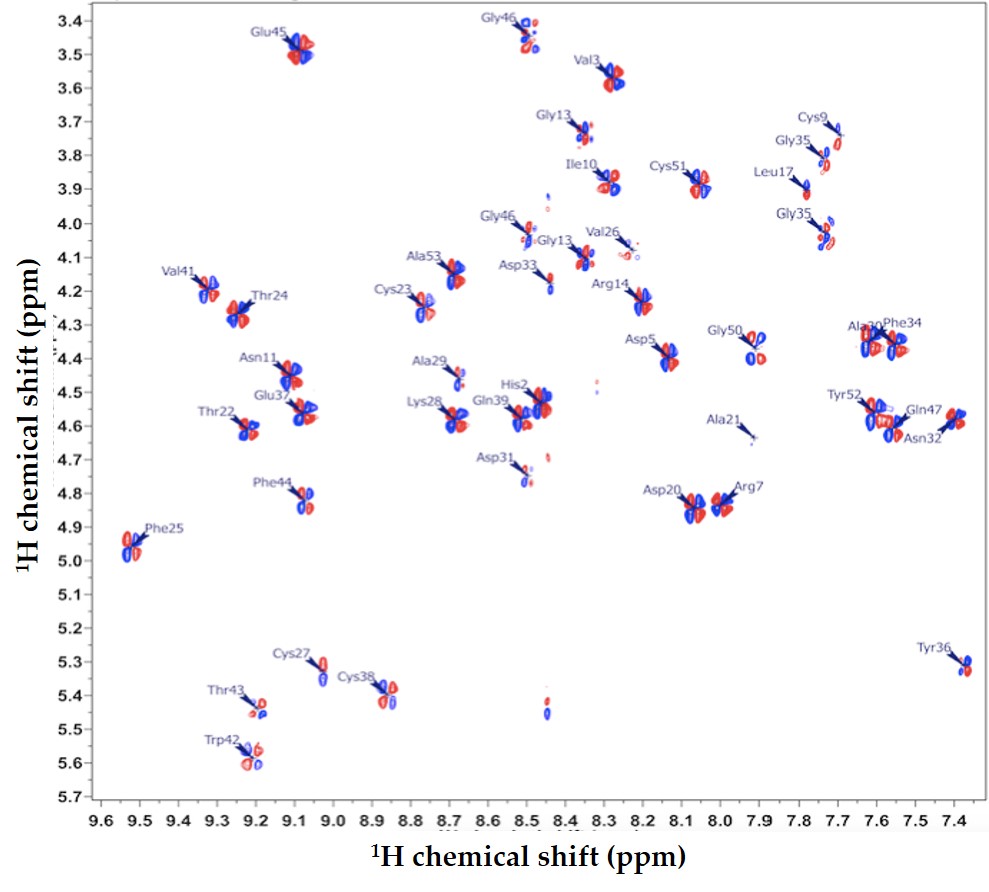


# Figure S5. ^1^H 500MHz 2D-NMR spectra of NvCI.

Spectra were obtained in 20mM sodium phosphate (pH 6.5). The expanded amide region of the DQF-COSY 2D proton/proton spectrum, at 25°C, in the presence of 10% D_2_O, is shown. Provisional cross-peak assignment was performed as indicated in Methods.


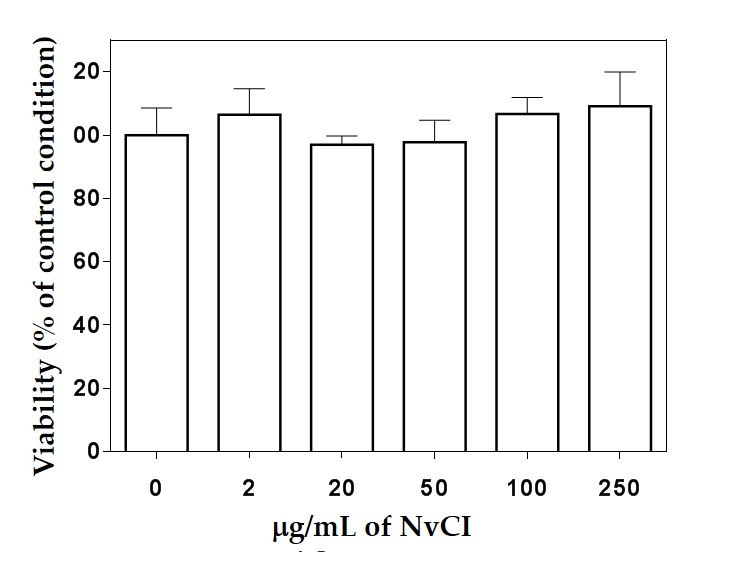


# Figure S6. Effects of NvCI on cytotoxicity/cell viability.

Cell viability of HepG2 cells after 72 h incubation with NvCI at various concentrations (2 to 250 µg/ml). Untreated cells were used as controls. Data represent the mean and standard error of the mean (SEM) of three independent experiments.

#
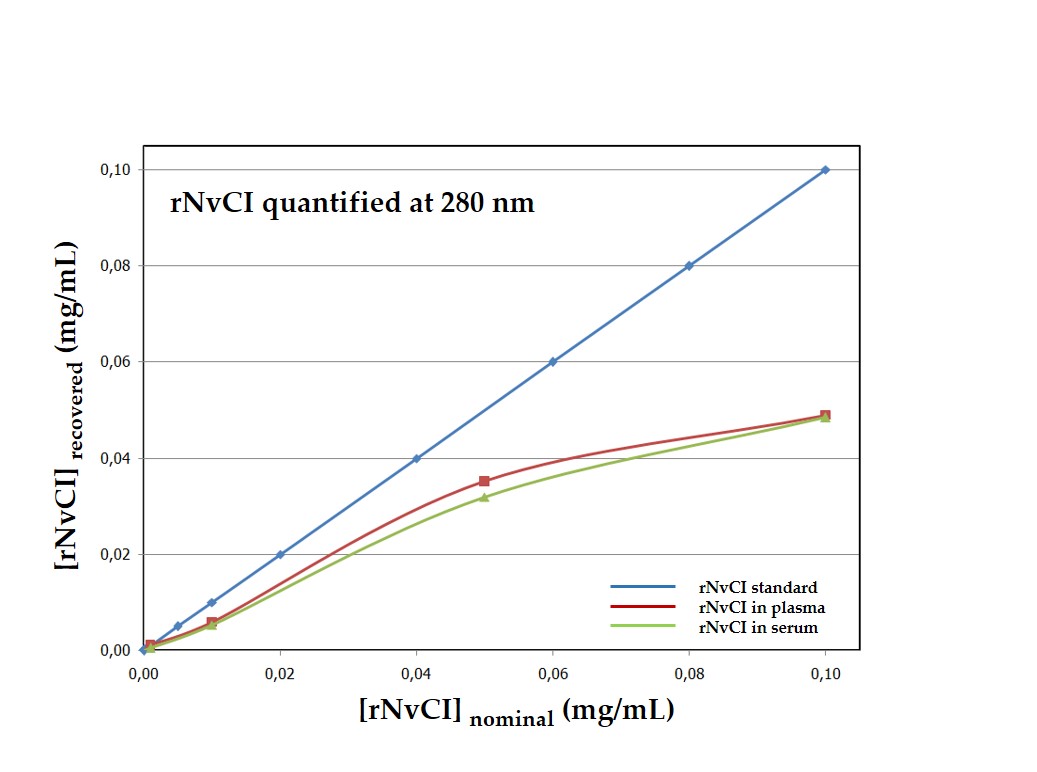
Figure S7. Calibration plot of the analytical quantitation of rNvCI incubated in blood plasma or serum, after spin-micro affinity capture followed by fraction recovery and HPLC analysis.

100 μL of distinct rNvCI samples were mixed with human blood plasma or serum and brought to concentrations from 0.0001 to 0.5mg/mL, the mix incubated for 1, 3 or 9h at 37°C, passed through a 100 μL Sepharose-CL-4B-CPA1 affinity column and, after washes with buffer, each main fraction recovered with 115 uL of TFA at 0.5%. Samples were loaded on a 5 μm HPLC-C4 reversed-phase column, equilibrated with 0.1% TFA, elution performed with an acetonitrile gradient and release and quantitation were followed at 280 and 214nm. The plots displayed in this figure only refer to samples without incubation (standard) or after incubation for 3h at 37°C before fractionation, at different NvCI concentrations.


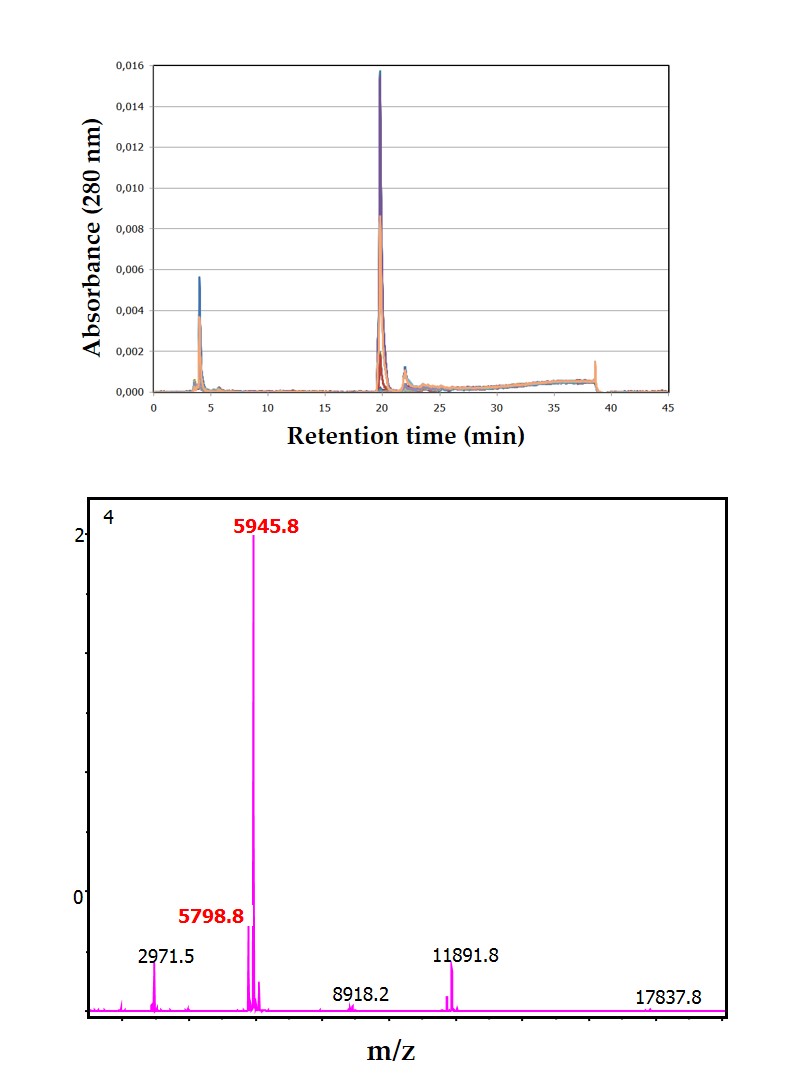


# Figure S8. Analysis of homogeneity and state of rNvCI by HPLC (top) and MALDI-TOF MS (bottom), after incubation and recovery from human blood plasma and serum.

The experimental conditions were described in the legend of Fig. S7, particularly for HPLC. The figure displays the experiment performed at the particular concentration of 0.05mg/mL of rNvCI in human plasma, after incubation for 9h at 37°C, affinity capture in the micro-spin-CPA1 column and acid release. With 10 μL remaining from the collected fraction from the affinity chromatography, a MALDI-TOF MS analysis was performed after mixing with matrix and drying, in a Bruker Extreme spectrometer.


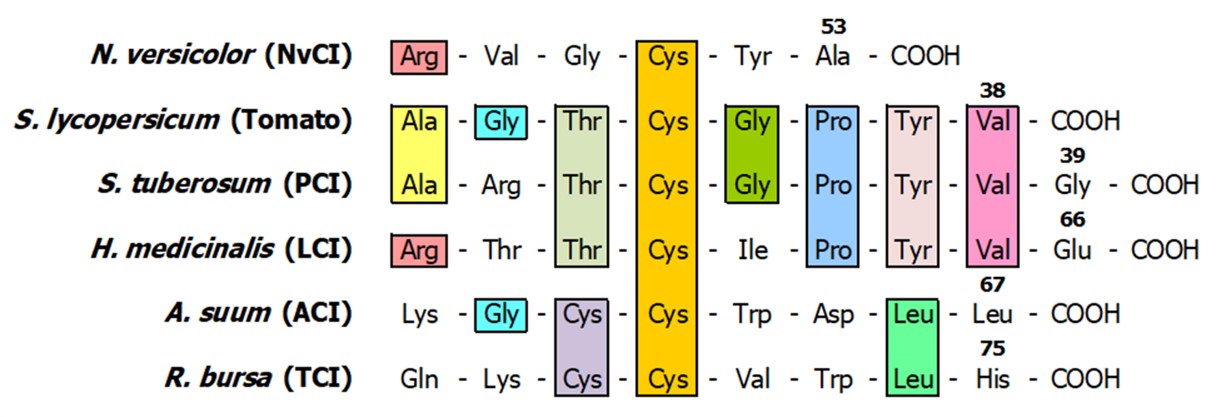


#

# Figure S9. Comparison of the C-terminal amino acid sequences of NvCI and other proteinaceous MCP inhibitors.

The inhibitors from different animal or vegetal species were aligned through the fitting of the sequences at the last cysteine residue, which is the expected boundary between the proteinaceous globular core and the C-tail.
