# Supplementary figures and images for: Characterization, Recombinant Production and Structure-Function Analysis of NvCI, A Picomolar Metallocarboxypeptidase Inhibitor from the Marine Snail Nerita versicolor
Source: Mar Drugs. 2019 Aug 29;17(9):511. doi: 10.3390/md17090511 (PMC6780499; doi:10.3390/md17090511)

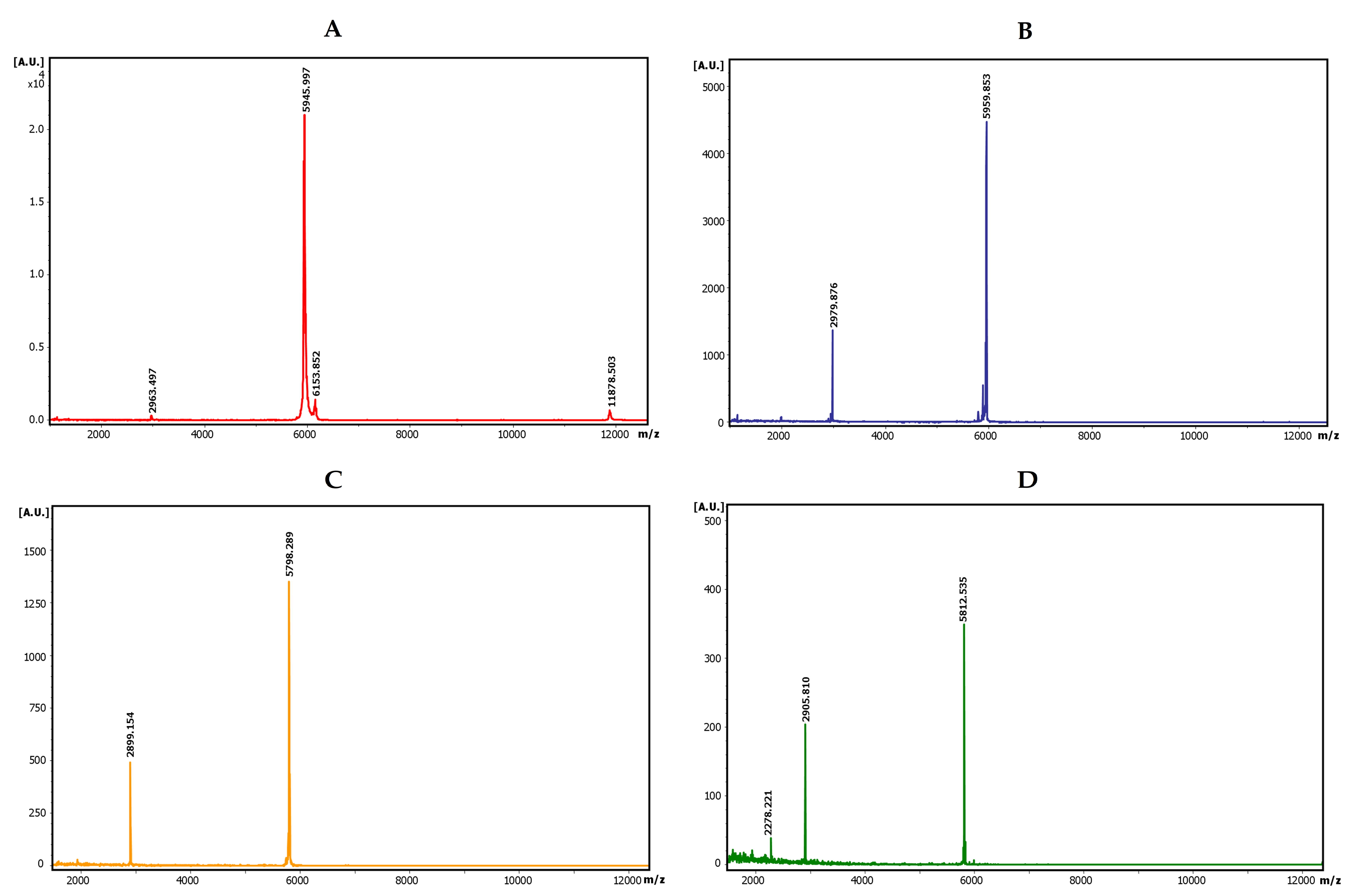

Supplement: Supplementary file 1 [file marinedrugs-17-00511-s001.zip › GCovaleda.MD-SupplemFile&HighResolFigs.25Aug/GCovaleda.MD-SupplFigs.300 dpi.2019VIII25/Figure S1.tif]

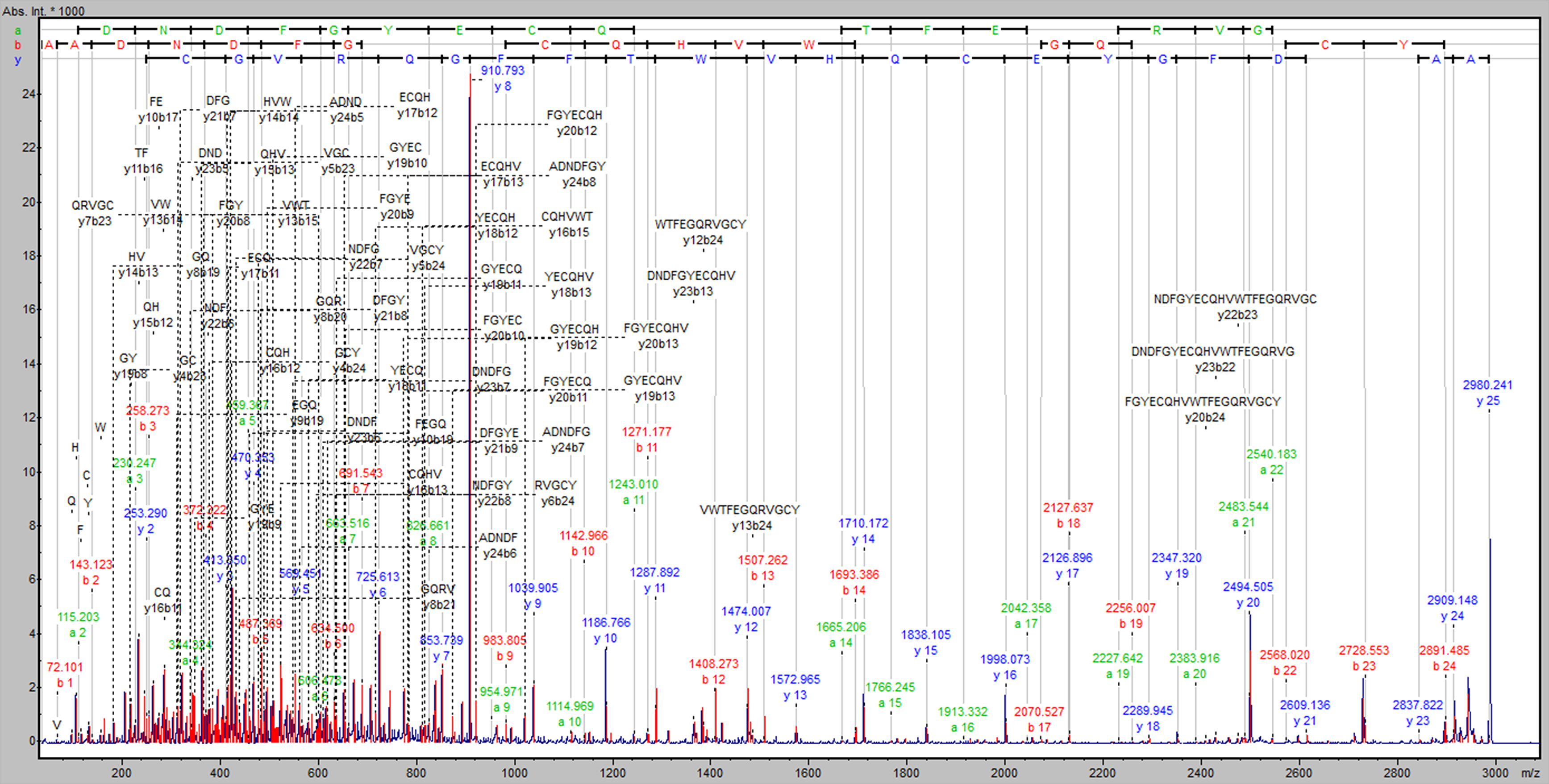

Supplement: Supplementary file 1 [file marinedrugs-17-00511-s001.zip › GCovaleda.MD-SupplemFile&HighResolFigs.25Aug/GCovaleda.MD-SupplFigs.300 dpi.2019VIII25/Figure S2.tif]

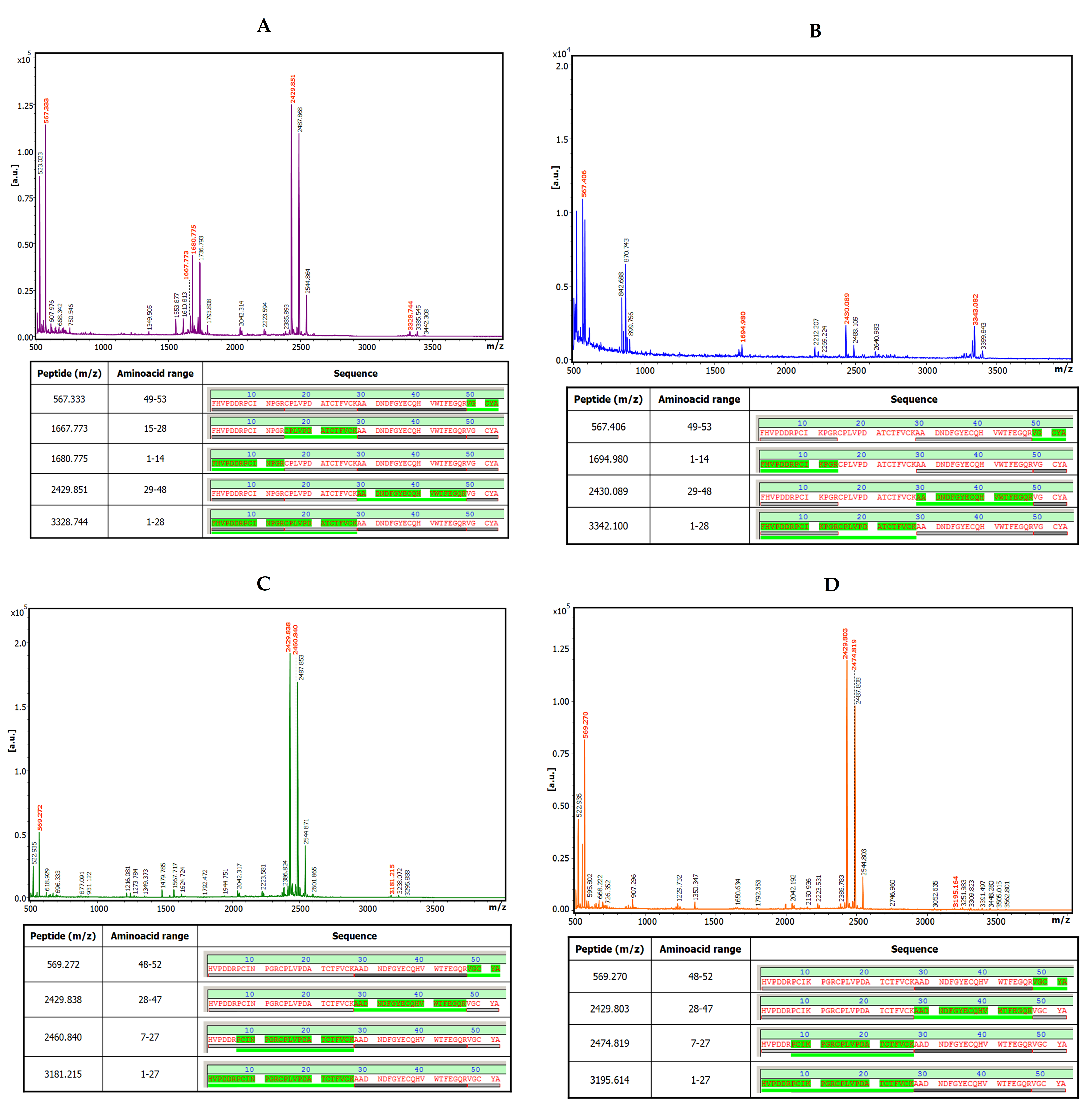

Supplement: Supplementary file 1 [file marinedrugs-17-00511-s001.zip › GCovaleda.MD-SupplemFile&HighResolFigs.25Aug/GCovaleda.MD-SupplFigs.300 dpi.2019VIII25/Figure S3.tif]

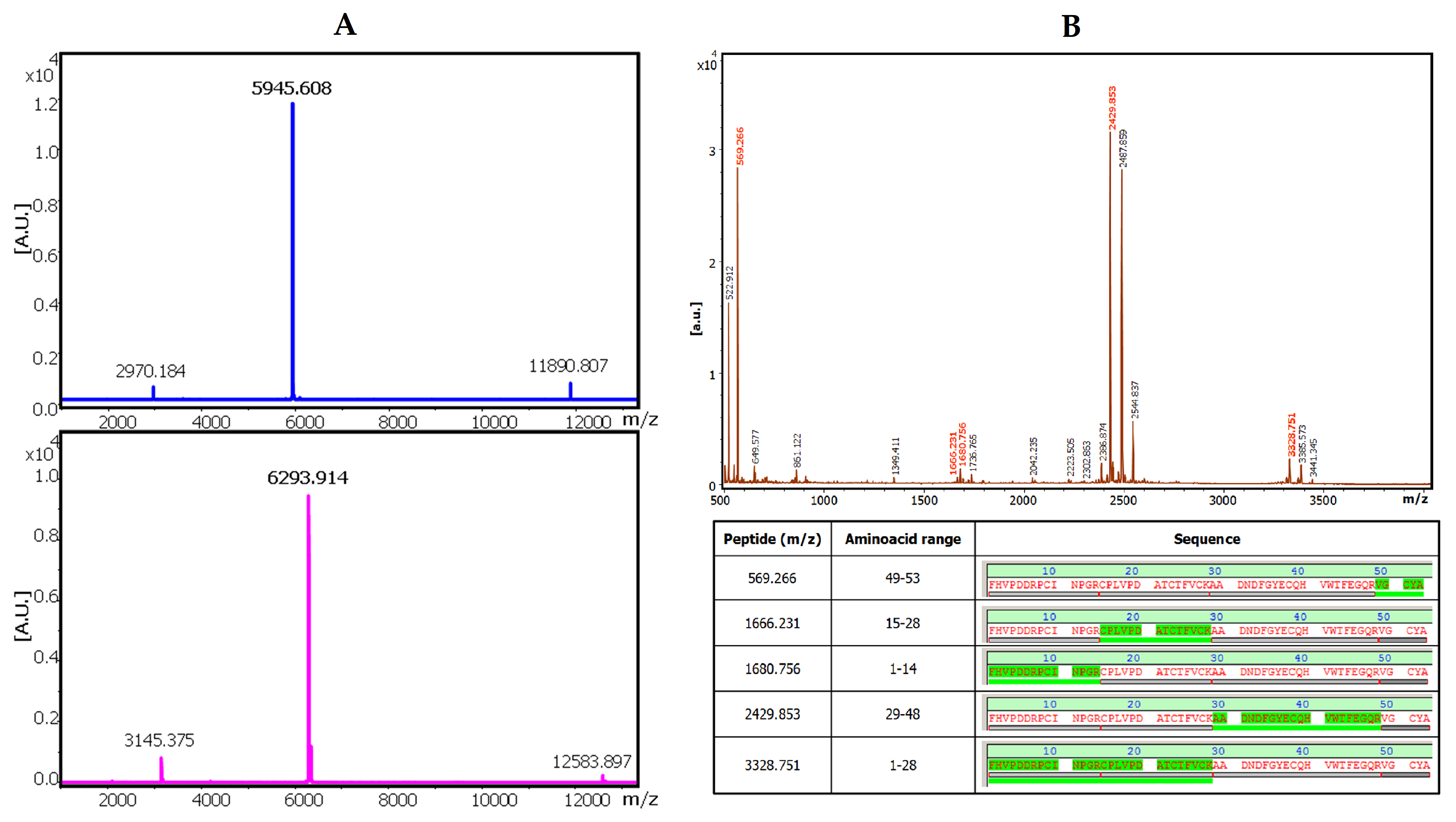

Supplement: Supplementary file 1 [file marinedrugs-17-00511-s001.zip › GCovaleda.MD-SupplemFile&HighResolFigs.25Aug/GCovaleda.MD-SupplFigs.300 dpi.2019VIII25/Figure S4.tif]

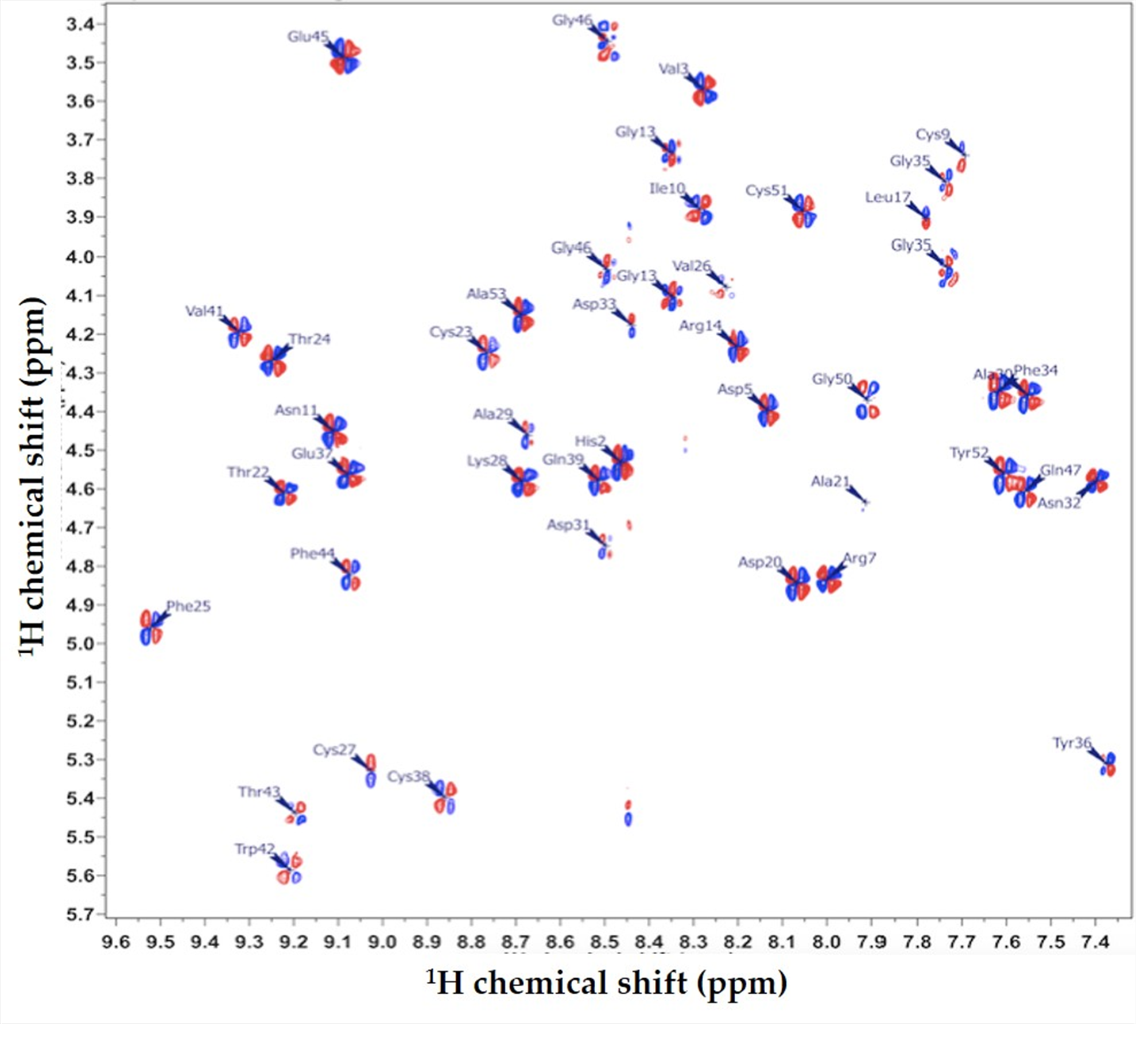

Supplement: Supplementary file 1 [file marinedrugs-17-00511-s001.zip › GCovaleda.MD-SupplemFile&HighResolFigs.25Aug/GCovaleda.MD-SupplFigs.300 dpi.2019VIII25/Figure S5.tif]

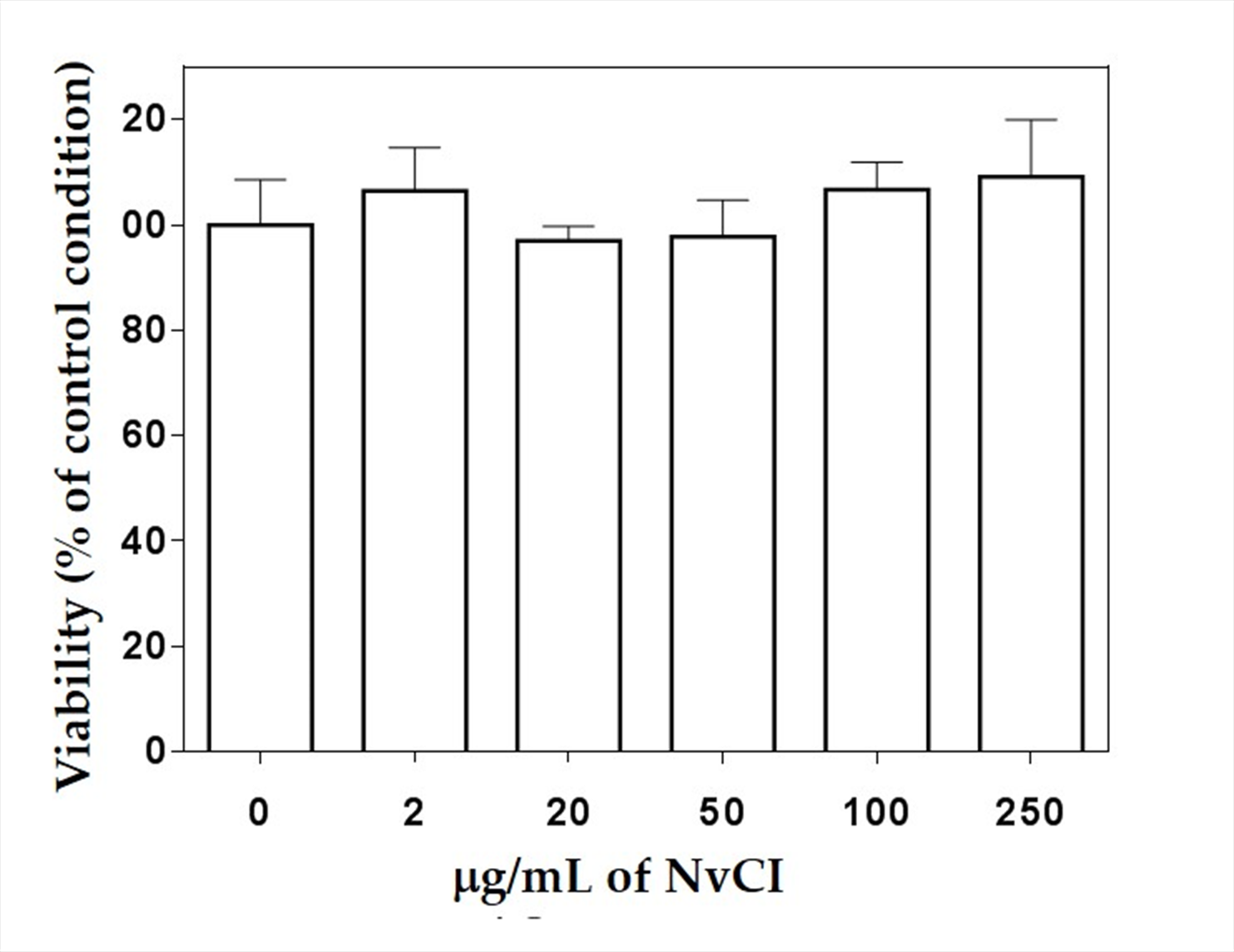

Supplement: Supplementary file 1 [file marinedrugs-17-00511-s001.zip › GCovaleda.MD-SupplemFile&HighResolFigs.25Aug/GCovaleda.MD-SupplFigs.300 dpi.2019VIII25/Figure S6.tif]

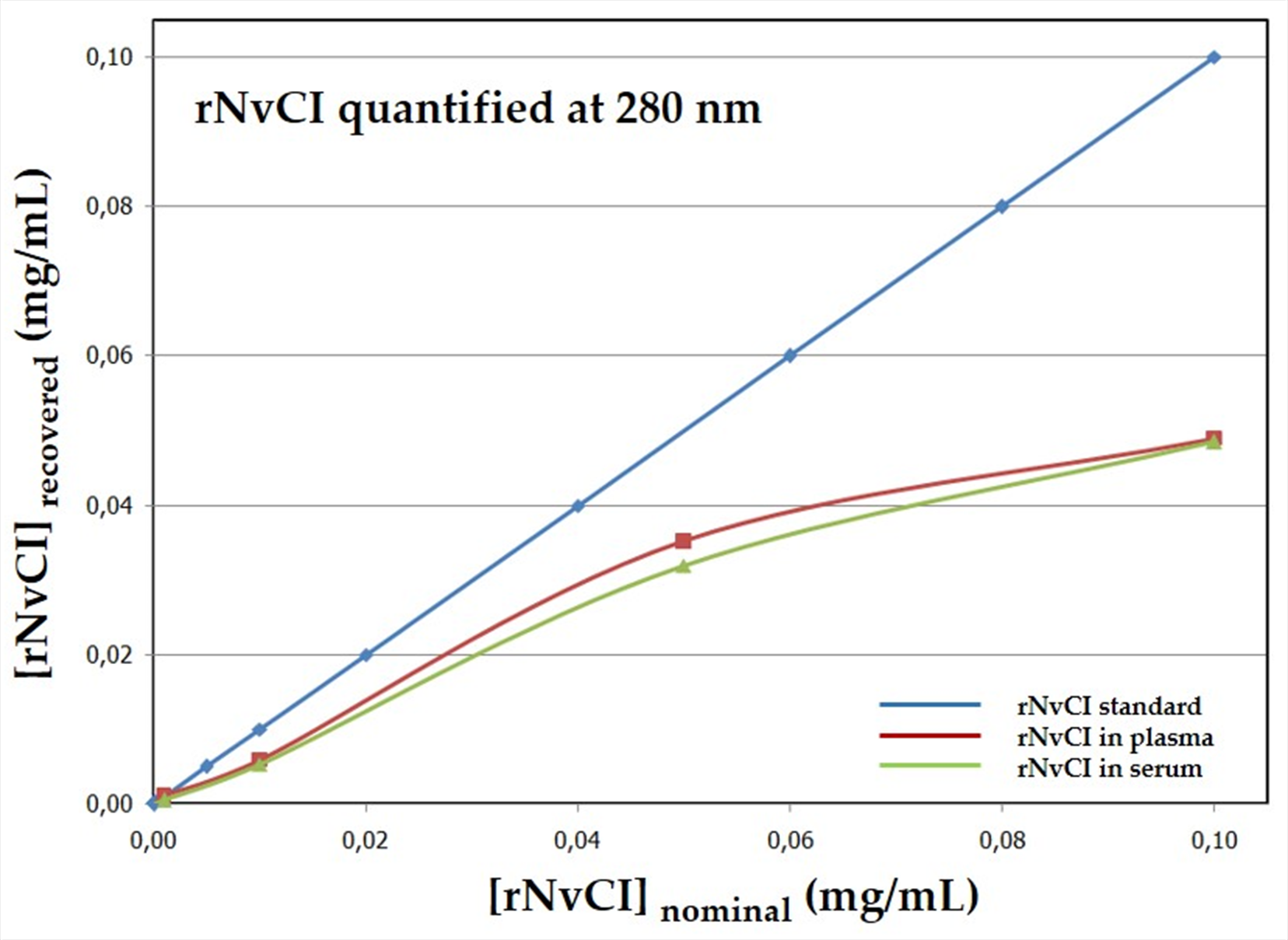

Supplement: Supplementary file 1 [file marinedrugs-17-00511-s001.zip › GCovaleda.MD-SupplemFile&HighResolFigs.25Aug/GCovaleda.MD-SupplFigs.300 dpi.2019VIII25/Figure S7.tif]

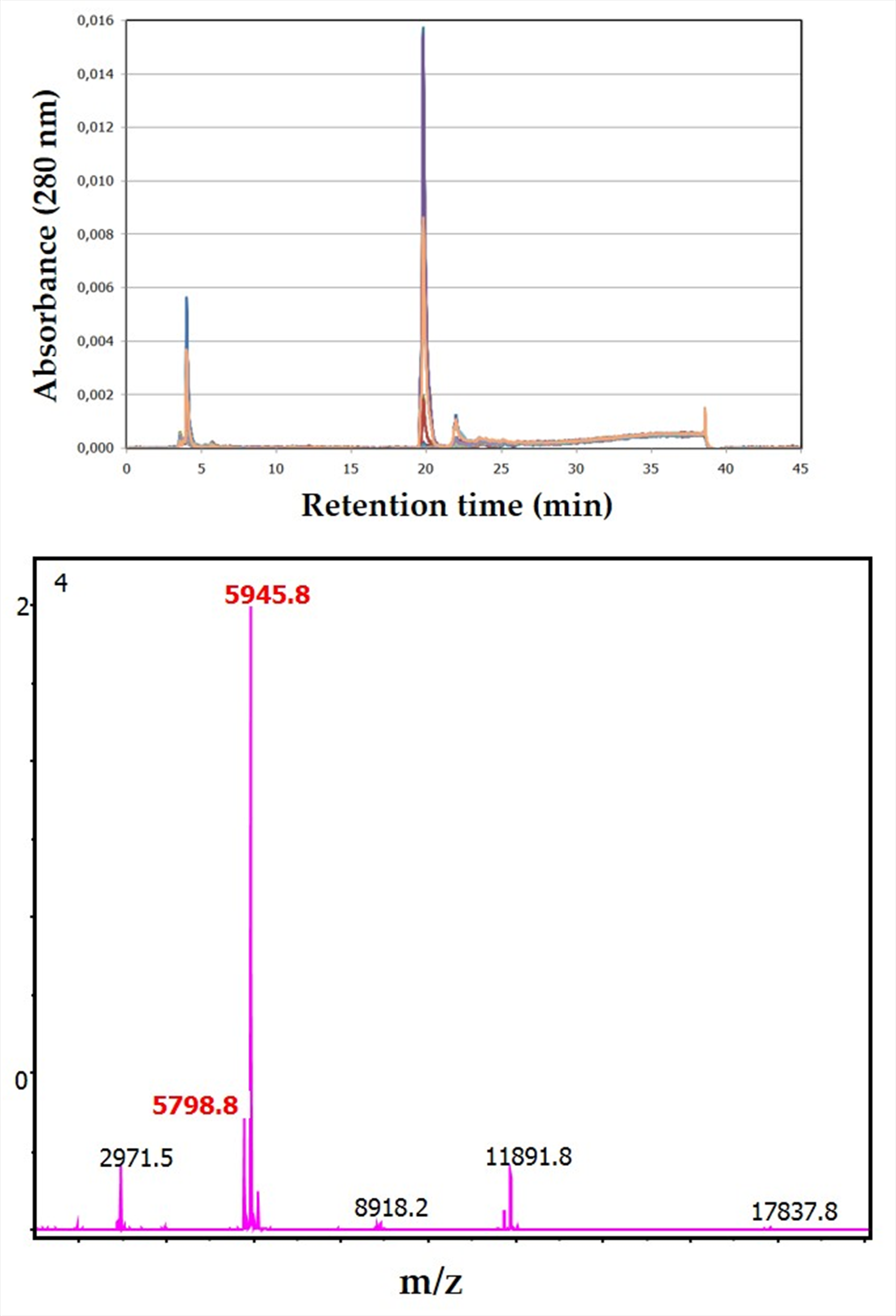

Supplement: Supplementary file 1 [file marinedrugs-17-00511-s001.zip › GCovaleda.MD-SupplemFile&HighResolFigs.25Aug/GCovaleda.MD-SupplFigs.300 dpi.2019VIII25/Figure S8.tif]

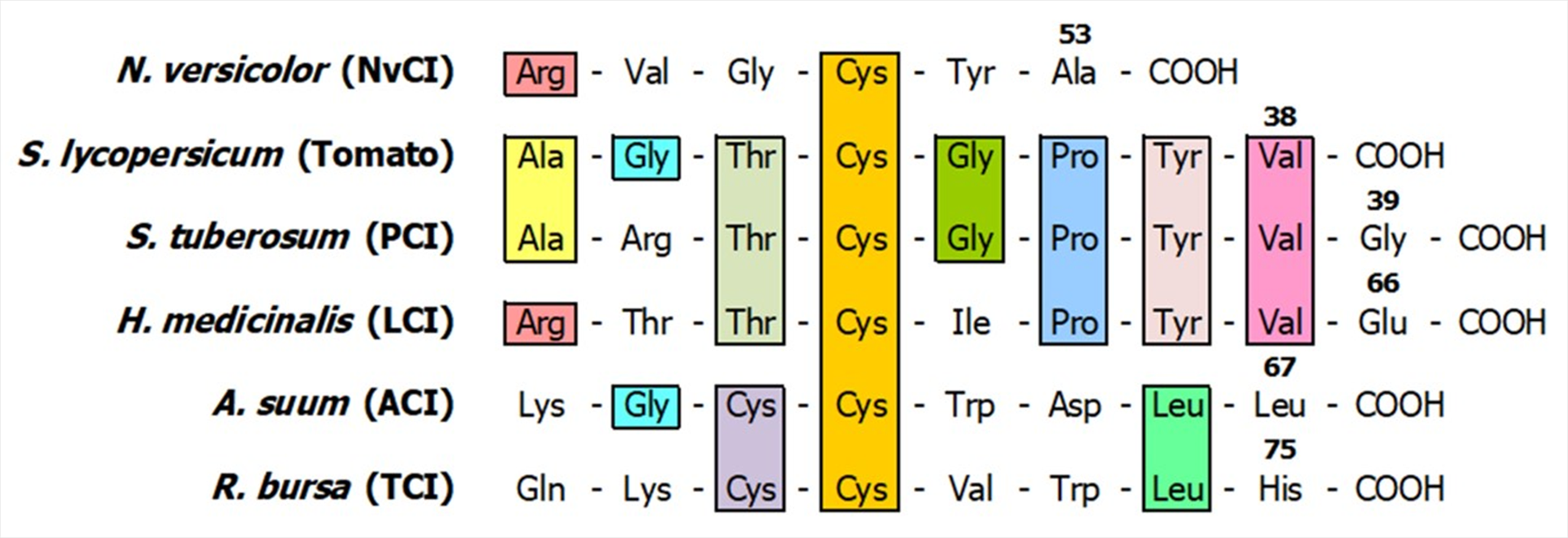

Supplement: Supplementary file 1 [file marinedrugs-17-00511-s001.zip › GCovaleda.MD-SupplemFile&HighResolFigs.25Aug/GCovaleda.MD-SupplFigs.300 dpi.2019VIII25/Figure S9.tif]
